# Distinct Roles of Nrf1 and Nrf2 in Monitoring the Reductive Stress Response to Dithiothreitol (DTT)

**DOI:** 10.3390/antiox11081535

**Published:** 2022-08-07

**Authors:** Reziyamu Wufuer, Zhuo Fan, Jianxin Yuan, Ze Zheng, Shaofan Hu, Guiyin Sun, Yiguo Zhang

**Affiliations:** 1 Bioengineering College, Chongqing University, No. 174 Shazheng Street, Shapingba District, Chongqing 400044, China; 2 Chongqing University Jiangjin Hospital, School of Medicine, Chongqing University, No. 725 Jiangzhou Avenue, Dingshan Street, Jiangjin District, Chongqing 402260, China; 3 The Laboratory of Cell Biochemistry and Topogenetic Regulation, College of Bioengineering and Faculty of Medical Sciences, Chongqing University, No. 174 Shazheng Street, Shapingba District, Chongqing 400044, China

**Keywords:** Nrf1, Nrf2, redox, oxidative stress, reductive stress, endoplasmic reticulum stress, antioxidant

## Abstract

Transcription factor Nrf2 (nuclear factor, erythroid 2-like 2, encoded by *Nfe2l2*) has been accepted as a key player in redox regulatory responses to oxidative or reductive stresses. However, relatively little is known about the potential role of Nrf1 (nuclear factor, erythroid 2-like 1, encoded by *Nfe2l1*) in the redox responses, particularly to reductive stress, although this ‘fossil-like’ factor is indispensable for cell homeostasis and organ integrity during the life process. Herein, we examine distinct roles of Nrf1 and Nrf2 in monitoring the defense response to 1,4–dithiothreitol (DTT, serving as a reductive stressor), concomitantly with unfolded protein response being induced by this chemical (also defined as an endoplasmic reticulum stressor). The results revealed that intracellular reactive oxygen species (ROS) were modestly increased in DTT-treated wild-type (*WT*) and *Nrf1α^−/−^* cell lines, but almost unaltered in *Nrf2^−/−^*^Δ^*^TA^* or *caNrf2*^Δ*N*^ cell lines (with a genetic loss of transactivation or *N*-terminal Keap1-binding domains, respectively). This chemical treatment also enabled the rate of oxidized to reduced glutathione (i.e., GSSG to GSH) to be amplified in *WT* and *Nrf2^−/−^*^Δ*TA*^ cells, but diminished in *Nrf1α^−/−^* cells, along with no changes in *caNrf2*^Δ*N*^ cells. Consequently, *Nrf1α^−/−^*, but not *Nrf2^−/−^*^Δ*TA*^ or *caNrf2*^Δ*N*^, cell viability was reinforced by DTT against its cytotoxicity, as accompanied by decreased apoptosis. Further experiments unraveled that Nrf1 and Nrf2 differentially, and also synergistically, regulated DTT-inducible expression of critical genes for defending against redox stress and endoplasmic reticulum stress. In addition, we also identified that Cys342 and Cys640 of Nrf1 (as redox-sensing sites within its *N*-glycodomain and DNA-binding domain, respectively) are required for its protein stability and transcription activity.

## 1. Introduction

In order to maintain cell redox homeostasis and organ integrity during the healthy life process, almost all cellular life forms have evolutionally established a set of proper defense mechanisms in response to a variety of challengeable (oxidative or reductive) stresses. Substances such as scavengers, blockers and repair agents of free radicals and reactive oxygen species (ROS) are collectively named antioxidants. Of note, not all reductants have antioxidant properties, because only those that can scavenge free radicals and ROS are de facto antioxidants. Recently, thiol compounds have been proved to protect the mitochondria from oxidative stress and relevant damage, which is attributed to their ability to scavenge oxygen and nitrogen free radicals [1,2,3]. Among them, *1*,*4-dithiothreitol* (DTT) is a well-known compound, because its structure is similar to that of reduced glutathione (GSH), so that it is often used to preserve the reductive state of sulfhydryl (-SH) groups in many proteins and assist in stabilizing their functional resilience. In fact, it is plausible that a reducing agent with antioxidant properties prevents intracellular inflammatory response [4]. DTT can prevent the oxidation of protein cysteine residues, but also interferes with the catalytic role of disulfide isomerase in disulfide bond formation of proteins. From this, DTT is hence reasoned to be a stimulator of endoplasmic reticulum (ER) stress [5]. Additional ROS, including DTT-relevant free radicals, are also produced to certain degrees. For instance, molecular oxygen is reduced to superoxide free radicals and hydrogen peroxide. Particularly, in the presence of trace metals (e.g., copper and iron), it is reduced to hydroxyl free radicals, resulting in DNA damage and cell apoptosis [6]. Such a paradoxical phenomenon, which not only has antioxidant properties, but also can induce reducing stress only when it is excessive, is worth exploring for the in-depth study of DTT [7]. Importantly, although DTT has been reported as a treatment-oriented method for some syndromes and complications [8,9], there are few studies on its application in liver cancer.

The adaptive reprogrammings are determined predominantly by those specified transcription factor-regulated gene expression networks at distinct layers, so as to give rise to a variety of stress responses to adverse external pressures or excessive demands [10,11]. As we know, such metabolic reprogramming, especially during the development and progression of cancer, is largely dependent on oxidative and reductive stress [12]. Within such multi-hierarchical regulatory networks, the Cap’n’collar (CNC) basic region-Leucine zipper (bZIP) family of transcription factors plays a vital role in maintaining robust redox homeostasis in the internal environments. Two principal CNC-bZIP family members, Nrf1 (nuclear factor, erythroid 2-related factor 1, encoded by *Nfe2l1*) and Nrf2 (nuclear factor, erythroid 2-related factor 2, encoded by *Nfe2l2*), enable the cellular resistance to challenging redox stresses by mediating the proper expression of those cytoprotective genes driven by antioxidant/electrophile response elements (AREs/EpREs) in their promoter regions. Although both factors are highly homologous, distinctions between Nrf1 and Nrf2 in their structures and subcellular locations determine their potential differences and overlaps in their physiobiological functions, which is a mutual adjustment relationship of ‘opposition and unity’ [13].

In this field, Nrf2 has been preferentially accepted as a master regulator of those antioxidant, detoxification, and cytoprotective genes in response to oxidative or reductive stresses [11,14,15,16,17], albeit a matter of fact that Nrf1, rather than Nrf2, is indispensable for cell homeostasis and organ integrity during normal development and growth, as well as adult life process [10]. This is possibly owing to the acute emergent response mediated by transcriptional activity of Nrf2 that is negatively regulated by a thiol-enriched electrophile sensor Keap1 (Kelch-like ECH-associated protein 1), which is attributable to their direct interaction enabling this water-soluble Nrf2 factor to be sequestered within the cytoplasmic subcellular compartments and targeted for its ubiquitination by Cullin 3-based E3 ubiquitin ligase, leading to its protein turnover by the ubiquitin-proteasomal degradation pathway. Only upon stimulation by redox electrophile or nucleophile stress, this thiol-based Keap1 is activated so as to allow Nrf2 to be released and then translocated into the nucleus, resulting in transactivation of target genes. By contrast, the membrane-bound Nrf1 is highly conserved as a ‘living fossil’ of organismal evolution from marine bacteria to mammals [18]. The nature-selected Nrf1 possesses a unique intrinsic characteristic to fulfill its special indispensable physiobiological functions that cannot be compensated by Nrf2 and other homologous factors [10]. It is of crucial significance to note the fact that *Nrf1^−/−^* cell lines and relevant model animals have suffered from severe endogenous oxidative stress, as manifested by obvious pathological phenotypes, one of which is exemplified by resembling human non-alcoholic steatohepatitis (NASH) and ultimate malignance to hepatoma. This is a rare case of the few typical cell-autonomous defects resulting from spontaneous oxidative stress, as far as we know in the current literature [10,14].

The unique functioning of Nrf1 is dictated by its original transmembrane topobiology, with specific post-translational modification and selective proteolytic processing during dynamic dislocation from the endoplasmic reticulum (ER) across membranes to enter extra-ER cyto/nucleoplasmic subcellular compartments before gaining access to its cognate genes [10]. Thereby, Nrf1 has been identified as an important ER sensor for changes in intracellular redox, glucose, proteins and lipids (including cholesterol) [19,20]. As we know, the ER is a key dynamic organelle involved in the control of eukaryotic cell function. Compared with the cytosol compartments, the ER luminal environment is the most oxidative in cells, and this phenomenon is considered to be conducive to the formation of disulfide bonds required for proper protein folding. The unbalanced changes in this environmental factor can lead to the destruction of ER homeostasis, which is usually called ER stress. Otherwise, it contains lower amounts of glutathione, although it has higher substrate affinity. Accordingly, this is considered to be one of the key sites of oxidative stress; such oxidative conditions are revealed to be of crucial importance to reduction reactions in the ER. Still, it is also reported that possible reduction stress may exist only temporarily and locally in the oxidative environment of ER. However, the threshold of its generation or changes remains elusive to date, although it has been shown that the steady state of ER may be more easily disturbed [21]. Our previous work demonstrated differential and integral contributions of Nrf1 and Nrf2 to coordinately mediating distinct responsive gene expression profiles to the ER stressor tunicamycin (TU) [20] and pro-oxidative stressor *tert*-butylhydroquinone (*t*BHQ) [22]. Recently, Nrf1, rather than Nrf2, was further identified as an indispensable redox-determining factor for mitochondrial homeostasis, in addition to the ER-associated proteostasis, by integrating multi-hierarchical responsive signaling pathways through nuclear respiratory gene controls towards mitochondrially located gene regulatory networks [23]. 

Herein, we found distinctive roles of Nrf1 and Nrf2 in synergistically monitoring the defense response to DTT as a reductive stressor, concomitantly with unfolded protein response being induced by this chemical (also serving as an ER stressor). Further evidence has been presented, revealing that intracellular reactive oxygen species (ROS) were modestly increased in DTT-treated wild-type (*WT*) and *Nrf1α^−/−^* cell lines, but almost unaltered in *Nrf2^−/−^*^Δ*TA*^ or *caNrf2*^Δ*N*^ cell lines (with a genetic loss of their transactivation or *N*-terminal Keap1-binding domains, respectively). This treatment also enabled the rate of oxidized to reduced glutathione (i.e., GSSG to GSH) to be amplified in *WT* and *Nrf2^−/−^*^Δ*TA*^ cells, but substantially diminished in *Nrf1α^−/−^* cells, along with no changes in *caNrf2*^Δ*N*^ cells. This finding indicates that a potential reductive stress is also induced, possibly by aberrant accumulation of hyperactive Nrf2 in *Nrf1α^−/−^* cells, apart from its severe endogenous oxidative stress. Consequently, *Nrf1α^−/−^*, but not *Nrf2^−/−^*^Δ*TA*^ or *caNrf2*^Δ*N*^, cell viability was reinforced by DTT against its cytotoxicity, as accompanied by decreased apoptosis. Lastly, we have also identified that Cys342 and Cys640 of Nrf1 (as redox-sensing sites situated within its *N*-glycosylated transactivation domain and its DNA-binding basic region, respectively) are required for this CNC-bZIP protein stability and its transcription activity.

## 2. Materials and Methods

### 2.1. Experimental Cell Lines and Culture

Human hepatocellular carcinoma HepG2 cells (i.e., *WT*) were obtained from the American Type Culture Collection (ATCC, Manassas, VA, USA). Three HepG2-derived knockout cell lines, *Nrf1α^−/−^*, *Nrf2^−/−^*^Δ*TA*^, and *caNrf2*^Δ*N*^ (with constitutive activation of *Nrf2*), were established in our laboratory as previously described by Qiu et al. [24]. Notably, the fidelity of HepG2 cells had been confirmed by authentication profiling and STR (short term repeat) typing map (Shanghai Biowing Applied Biotechnology Co., Ltd., Shanghai, China). All of these cells were incubated at 37 °C in 5% CO_2_, and allowed to grow in DMEM supplemented with 25 mmol/L glucose (Gibco, Stanford, CA, USA), 10% (*v*/*v*) FBS (Gibco, Stanford, CA, USA) and 100 units/mL penicillin-streptomycin (MACKLIN, Shanghai, China).

### 2.2. Reagents and Antibodies

The chemical *(2S*, *3S)-1*,*4-Bis-sulfanylbutane-2*,*3-diol* (DTT, CAS No. 3483-12-3) was obtained from Sigma; it is a strong reductant with the chemical formula *C*_4_*H*_10_*O*_2_*S*_2_ and *MW* of 154.25 g/mol. Its reducibility is largely due to the conformational stability after oxidation (containing disulfide bond). In this experiment, 3.09 g DTT powder was completely dissolved in 20 mL 0.01 M sodium acetate to obtain 1 M DTT stock solution, which was packed and stored at −20 °C before use. 

Specific antibody against Nrf1 was made in our own laboratory [25]. All nine distinct antibodies against Nrf2 (ab62352), GCLC (ab207777), GCLM (ab126704), HO-1 (ab52947), GPX1 (ab108427), XBP1 (AB109221), ATF4 (ab184909), ATF6 (ab227830) and P4HB (ab137180) were obtained from Abcam (Cambridge, UK). The first three antibodies against TALDO (D623398), GSR (D220726) and NQO1 (D26104) were purchased from Sangon Biotech (Shanghai, China). The second three antibodies against BIP (bs-1219R), Chop (bs-20669R) and pIRE1 (bs-16698R) were from Bioss (Beijing, China). The third three antibodies against PSMB5 (A1975), PSMB6 (A4054), PSMB7 (A14771) were from ABclonal (Wuhan, China). Lastly, three antibodies to p-eIF2α (#5199) were from Cell Signaling Technology (Boston, MA, USA), p-PERK (sc-32577) from Santa CruZ (Santacruz, CA, USA), and β-actin (TA-09) from ZSGB-BIO (Beijing, China), respectively.

### 2.3. Cell Viability Assay

All four genotypic cell lines (*WT*, *Nrf1α^−/−^*, *Nrf2^−/−^*^Δ*TA*^ and *caNrf2*^Δ*N*^) were plated in 96-well plates at a density of 6 × 10^3^ cells per well, each treatment of which was repeated in six separated wells. After the cells completely adhered, they were treated with different concentrations of DTT (0.5, 1.0, 2.0, 3.0 or 4.0 mM) for 24 h or cultured with a single dose of 1 mM DTT for different lengths of time (0, 4, 8, 12, 16, 20 or 24 h). Additionally, MTT reagent (10 μL/well of 10 mg/mL stocked) was used to detect the cell viability.

### 2.4. Intracellular ROS Measurement

Equal numbers (3 × 10^5^ cells/well in 6-well plate) of experimental cells (*WT*, *Nrf1α^−/−^*, *Nrf2^−/−^*^Δ*TA*^ and *caNrf2*^Δ*N*^) were allowed to grow for 24 h. After reaching 80% confluence, the cells were treated with 1 mM DTT for distinct time periods (i.e., 0, 4, 12, 24 h) and then collected. The intracellular ROS were determined by flow cytometry, according to the instructions of the ROS assay kit (S0033S, Beyotime, Shanghai, China). Additionally, four different genotypic cells were also plated in 3.5-cm plates with 2.5 × 10^5^ cells/plate and treated as described above. Thereafter, the cells were cleaned with cold PBS buffer and then incubated with 10 μM of DCFH-DA staining solution (ROS assay kit) at 37 °C for 20 min, before being visualized under a fluorescence microscope.

### 2.5. Assay for the GSSG to GSH Ratio

All experimental cells were seeded in 6-well plates (4 × 10^5^ cells/well). The cells were then treated with 1 mM DTT for different lengths of time, and then collected, before being subjected to determination of the GSSG to GSH ratio according to the instructions of a T-GSH and GSSG assay kit (A061-1-2, Nanjing Jiancheng bioengineering institute, Nanjing, China). 

### 2.6. The Pulse-Chase Experiments for Nrf1 and Its Mutants with Distinct Trans-Activity 

Only four cysteine (Cys) residues of Nrf1 are in its distinct domains: Cys342 situated in its *N*-glycosylated NST domain, with two closer residues, Cys521 and Cys533, in its Neh6L domain that is highly homologous with Nrf2, and Cys640 placed in the basic DNA-binding region. They were subjected to site-directed mutagenesis into serine (*S*) residues, respectively. The resulting expression constructs for Nrf1 and its mutants were transfected into experimental cells, before the cells were treated with 50 μg/mL cycloheximide (CHX, a protein synthesis inhibitor) alone or together with the proteasome inhibitor MG132 (10 μM) for different periods of time (0, 0.25, 0.5, 1, 2 or 4 h). Thereafter, these protein samples were collected and then subjected to measurement of their half-lives. In addition, *trans*-activity of Nrf1 and its mutants was determined by the *pGL3-basic-6* × *ARE-Luc* reporter assay, as described previously [22]. 

### 2.7. Real-Time Quantitative PCR Analysis of Key Gene Expression

After all experimental cells reached 80% confluence, they were treated with 1 mM of DTT for indicated periods of time. Total RNAs were extracted and then subjected to reaction with a reverse transcriptase to synthesize the first strand of cDNAs. Subsequently, the mRNA levels of examined genes in different cell lines were determined by real-time quantitative PCR (RT-qPCR) with each indicated pair of their forward and reverse primers (as listed in Table 1). All experiments were carried out in the Go Taq real-time PCR detection systems with a CFX96 instrument. The resulting data were analyzed by the Bio-Rad CFX Manager 3.1 software.

### 2.8. Western Blotting Analysis of Key Protein Expression

Different genotypic cell lines were plated on 6-well plats and exposed to the indicated experimental conditions. The expression abundances of selected proteins were determined by Western blotting. Briefly, after quantitating total proteins in each sample with the BCA protein reagent (P1513-1, ApplyGen, Beijing, China), they were separated by SDS-PAGE gels (8% to 12% polyacrylamide) and then transferred onto PVDF membranes. After blocking in Tris-buffered saline containing 5% non-fat dry milk at room temperature for 1 h, the membranes were incubated with specific primary antibodies overnight at 4 °C. After washing with PBST, the blotted membranes were re-incubated with the secondary antibodies at room temperatures for 2 h. After imaging with Bio-Rad, the intensity of immunoblotted proteins was calculated by the Quantity One software 4.6.2, while β-actin was used as a loading control. 

### 2.9. Statistical Analysis

The data presented herein are shown as fold changes (*mean* ± *SD*) relative to controls, which were calculated from at least three independent experiments, each of which was performed in triplicate. Statistical significance was assessed by one-way ANOVA, and the Tukey’s post hoc test was used to determine the significance for all pairwise comparisons of interest. Differences between distinct treatments were considered to be statistically significant at *p* < 0.05 or *p* < 0.01.

## 3. Results 

### 3.1. Distinct Intervening Effects of DTT on Different Genotypic Cell Survival and Nrf1 and Nrf2 Expression 

**Figure 1:**
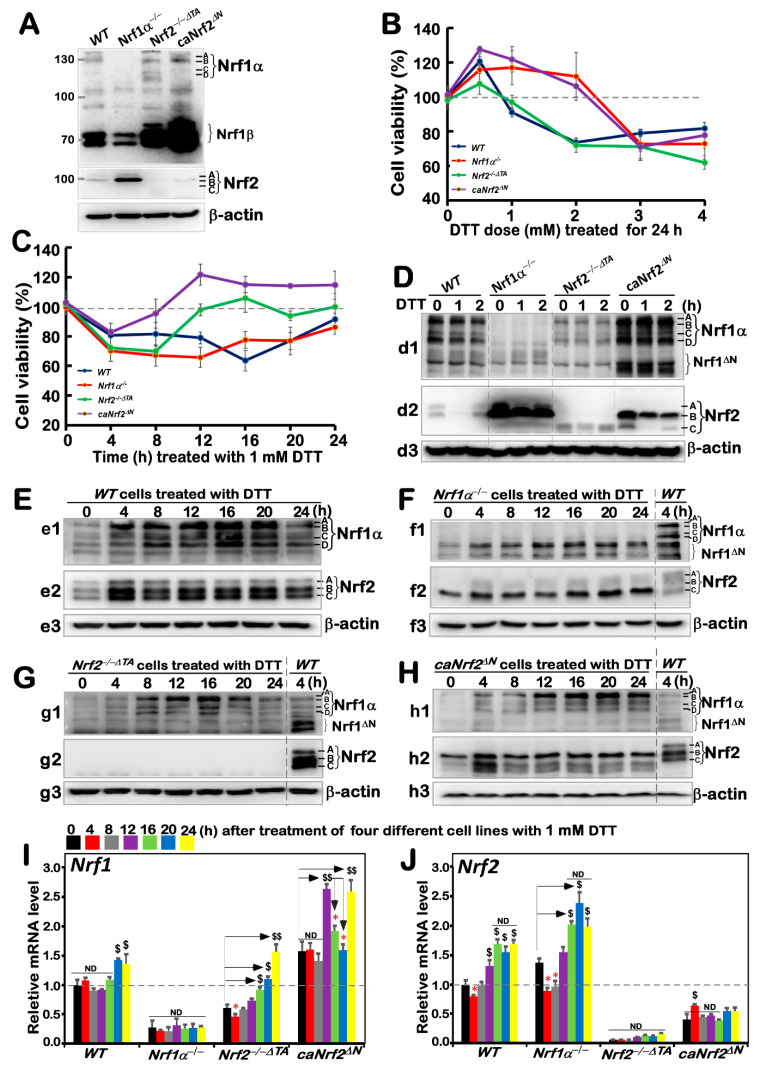
An internal regulatory relationship between Nrf1 and Nrf2 in mediating reductive stress response to DTT. (**A**) Distinct protein levels of Nrf1 and Nrf2 in four different genotypic cell lines *WT*, *Nrf1α^−/−^*, *Nrf2^−/−^*^△*TA*^ and *caNrf2*^△*N*^ were determined by Western blotting with their specific antibodies. (**B**,**C**) The viability of these cell lines was detected by the MTT-based assay, after they had been treated with different concentrations of DTT for 24 h (**B**) or treated with 1 mM of DTT for different lengths of time (**C**). (**D**–**H**) The Nrf1 (*d1*, *e1*, *f1*, *g1*, *h1*), Nrf2 (*d2*, *e2*, *f2*, *g2*, *h2*) and *β*-actin (*d3*, *e3*, *f3*, *g3*, *h3*) protein expression levels in four genotypic experimental cell lines following intervention with 1 mM of DTT for short times (i.e., 0, 1, 2 h) (**D**) and long times (i.e., 0, 4, 8, 12, 16, 20, 24 h) (**E**–**H**). (**I**,**J**) Both *Nrf1* and *Nrf2* mRNA expression levels in different cell lines that had been treated with 1 mM DTT for distinct time periods (i.e., 0, 4, 8, 12, 16, 20, 24 h) were examined by real-time qPCR. The data are representative of at least three independent experiments, each performed in triplicate. Significant increases (*$*, *p* < *0.05; $$*, *p* < *0.01*) and significant decreases (* *p* < *0.05*), in addition to non-significance (ND), were statistically determined when compared with the corresponding controls (measured at 0 h), respectively. All protein-blotted bands were also quantified by Quantity One 4.6.2 software, as showed in Figure S1.

Before this experiment, we verified the characteristic proteins of four cell lines with different genotypes, and confirmed them to be true, as reported previously [20,24] (Figure 1A). Besides, as mentioned by Xiang et al. [26], there were four major isoforms derived from human Nrf1α: A and B represent its full-length glycoprotein and deglycoprotein, respectively, while C and D denote two distinct lengths of its *N*-terminally truncated proteins. All these four Nrf1α-derived isoforms were completely deleted upon specific knockout of Nrf1α in *Nrf1α^−/−^* cells (Figure 1A), but still present in *WT*, *Nrf2^−/−^*^Δ*TA*^, and *caNrf2*^Δ*N*^ cell lines. Relatively, the short Nrf1β was obviously decreased in *Nrf1α^−/−^* cells, but significantly augmented in both *Nrf2^−/−^*^Δ*TA*^, and *caNrf2*^Δ*N*^ cell lines when compared to its equivalent in *WT* cells, indicating that basal abundances of Nrf1 and/or its processing may be monitored by Nrf2, besides itself. It is worth noting that Nrf2 was highly expressed in *Nrf1α^−/−^* cell lines, but completely abolished in *Nrf2^−/−^*^Δ*TA*^ cells. Overall, it is inferable that loss of Nrf1α or Nrf2 may lead to putative inter-regulatory changes in cognate gene expression profiles among these four cell lines, which were used in subsequent experiments.

Based on redox characteristics, a reducing compound, DTT, was employed to explore the effect of foreign substances on cell viability of different genotypes by MTT assay, so as to evaluate the formation of formazan precipitates with succinate dehydrogenase in the mitochondria of all living cells only. The results revealed that the viability of all cell lines decreased when they were intervened for 24 h by higher concentrations (3 to 4 mM) of DTT (Figure 1B), although lower concentrations (0.5–2 mM) of DTT caused modestly enhanced survival of *Nrf1α^−/−^* and *caNrf2*^Δ*N*^ cell lines. By contrast, a significant dose-dependent effect of DTT was manifested in *Nrf2^−/−^*^Δ*TA*^, similarly to that obtained from *WT* cells. Considering such different survival between cells along with the cytotoxicity of DTT, we selected a more appropriate dose at 1 mM of this compound to continue intervention of all experimental cell lines for distinct periods of time from 0 to 24 h (i.e., 0, 4, 8, 12, 16, 20 or 24 h). As shown in Figure 1C, the viability of all four cell lines was modestly decreased within 8 h of DTT intervention. Such decreased viability of *Nrf1α^−/−^* and *WT* cell lines was maintained for 20 h, after which they gradually recovered from intervention. By contrast, the resilience of *Nrf2^−/−^*^Δ*TA*^ and *caNrf2*^Δ*N*^ cell lines appeared to be strikingly recovered after 8 h to 12 h intervention with DTT, and then reached or exceeded their basal levels, respectively. However, the overall viability of these cell lines was not very significant, and thus, several time periods of DTT intervention were selected with the reference value for follow-up experiments to explore the regulatory differences between Nrf1 and Nrf2 in mediating the cellular reductive stress responses to intervention of this reducing compound. 

The short-term (i.e., 0, 1, 2 h) intervention of DTT did not cause a significant difference in Nrf1 abundances in each of the other three cell lines except *Nrf1α^−/−^* (Figure 1(d1)), while Nrf2 protein abundances were only modestly decreased in each of the other three cell lines except *Nrf2^−/−^*^Δ*TA*^ (Figure 1(d2), although Nrf1 and Nrf2 were rather highly expressed in *Nrf1α^−/−^* and *caNrf2*^Δ*N*^ cell lines), when compared with their respective basal levels (obtained at time point 0). Therefore, the time of intervention was extended from 4 h to 24 h, so as to gain the time-dependent effect of DTT on Nrf1 and Nrf2, as well on their targets. 

The resulting data are illustrated in Figure 1E, revealing significant increases in Nrf1α-derived isoforms, particularly its glycoprotein-A, with the extended time of DTT stimulation from 4 h to 20 h, by comparison with *WT_t0_* as the vehicle control. DTT-inducible expression of Nrf2 was also increased to a considerably higher level after 4 h of this treatment, and so higher expression was maintained to 24 h before stopping experiments. Such changes in Nrf1 and Nrf2 when *WT* cells were exposed to DTT for 4 h (i.e., *WT_t4_*) served as the ensuing parallel experimental controls. Next, examinations of DTT-treated *Nrf1c* cells unraveled that albeit this protein itself was completely lost, highly expressed Nrf2 was further enhanced to much higher levels than that obtained from the *WT_t4_* (Figure 1F). In *Nrf2^−/−^*^Δ*TA*^ cells, although basal expression of Nrf1 seemed weaker than that of *WT_t4_*, its DTT-inducible expression was substantially augmented (Figure 1G), specifically after this chemical intervention from 8 h to 20 h, whereas Nrf2 was totally abolished. In *caNrf2*^Δ*N*^ cells, the expression levels of Nrf1 and Nrf2 were also significantly incremented by DTT in a time-dependent manner (Figure 1H). All relevant immunoblots were quantitatively analyzed as shown in supplemental Figure S1.

Further examinations by RT-qPCR showed that DTT intervention of *WT* cells only caused a marginally slower increase in Nrf1 mRNA expression after 20 h stimulation, while Nrf2 mRNA levels were significantly incremented after only 12 h DTT treatment when compared to its basal levels (Figure 1I,J). In *Nrf1α^−/−^* cells, Nrf2 mRNA expression was also further augmented, although Nrf1 expression was largely abrogated. By contrast, although basal Nrf1 expression in *Nrf2^−/−^*^Δ*TA*^ cells was prevented, its DTT-stimulated expression was still enhanced after 16 h of this treatment (Figure 1I). However, much to our surprise, we found that although putative constitutive active protein of Nrf2 was present in *caNrf2*^Δ*N*^ cells, its mRNA expression levels were unaffected by DTT (Figure 1J), whereas conversely, a striking elevation of Nrf1 mRNA expression occurred after 12 h treatment. Overall, these findings demonstrate that a potential inter-regulatory relationship exists between Nrf1 and Nrf2, albeit under DTT-leading reductive stress conditions.

### 3.2. Differential Expression of Nrf1/2-Mediated Redox Responsive Genes Induced by DTT in Distinct Genotypic Cells

**Figure 2:**
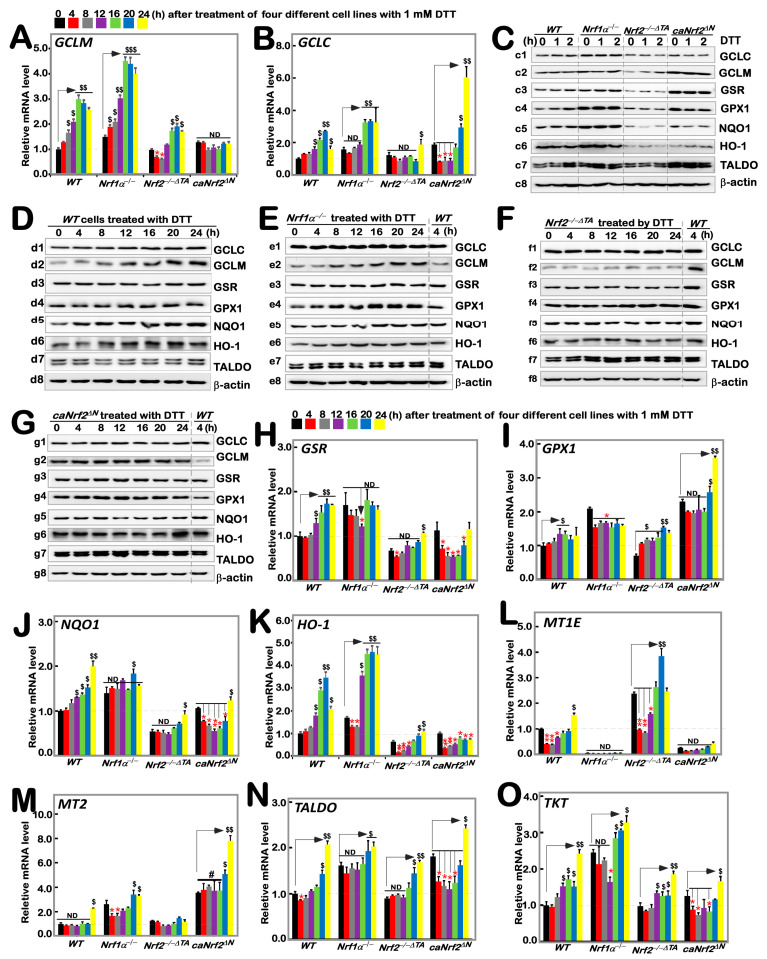
Distinct time-dependent expression of Nrf1/2-mediated redox response genes to DTT in different cell lines. Different genotypic cell lines *WT*, *Nrf1α^−/−^*, *Nrf2^−/−^*^△*TA*^ and *caNrf2*^△*N*^ were or were not treated with 1 mM DTT for 0 to 24 h, before both basal and DTT-inducible mRNA levels of all examined genes were determined by RT-qPCR. These genes included GCLM (**A**), GCLC (**B**), GSR (**H**), GPX1 (**I**), NQO1 (**J**), HO-1 (**K**), MT1E (**L**), MT2 (**M**), TALDO (**N**) and TKT (**O**). The resulting data were shown as fold changes (*mean* ± *SD*, *n* = *3* × *3*), which are representative of at least three independent experiments being each performed in triplicate. Significant increases (*$*, *p* < *0.05*; *$$*, *p* < *0.01*; *$$$*, *p* < *0.001*) and significant decreases (* p < *0.05*; ** p < *0.01*), in addition to the non-significance (ND or #), were statistically analyzed when compared with their corresponding controls (measured at 0 h), respectively. The up and down regulatory changes of different genes after 1 mM DTT intervention can be seen in Figure S5A. These experimental cells were or were not treated with 1 mM for short times (i.e., 0, 1, 2 h) (**C**) or long times (i.e., 0, 4, 8, 12, 16, 20, 24 h) (**D**–**G**), before basal and DTT-inducible protein abundances of GCLC (**c1**–**g1**), GCLM (**c2**–**g2**), GSR (**c3**–**g3**), GPX1 (**c4**–**g4**), NQO1 (**c5**–**g5**), HO-1 (**c6**–**g6**), TALDO (**c7**–**g7**) and TKT (**c8–g8**) were determined by Western blotting with indicated antibodies, whilst *β*-actin served as a loading control. The intensity of those immunoblots was also quantified by the Quantity One 4.6.2 software, as shown in Figure S2.

According to the results of transcriptome sequencing analysis of four genotypes of cells by Qiu et al., Nrf1 and Nrf2 have regulatory differences in the expression of GCLC, GCLM, GSR and other oxidative stress related genes [24]. Additionally, our previous study had shown that tBHQ, as a small molecule antioxidant, stimulates mRNA expression of some *ARE*-dependent genes downstream of Nrf1 and Nrf2 (such as *GCLM*, *GCLC*, *HO1*, etc.). Here, we examined whether these ARE-driven genes were affected by intervention of DTT as a reducing agent of sulfhydrylation. As anticipated, RT-qPCR results showed that significant time-dependent increases in DTT-inducible mRNA expression of *GCLM* and *GCLC* encoding two subunits of GSH synthesis rate-limiting enzyme are of crucial importance in the redox process [27] in DTT-treated *WT* cells (Figure 2A,B). Knockout of *Nrf1α^−/−^* cells caused a faster significant increase in DTT-inducible expression of *GCLM* from 4 h to its maximum at 16 h, which was then maintained to 24 h before stopping experiments (Figure 2A), while only a modest increase in *GCLC* expression was apparently lagged, occurring from 16 h stimulation by DTT (Figure 2B). By sharp contrast, *Nrf2^−/−^*^Δ*TA*^ cells only displayed a marginal increase in expression of *GCLM*, whereas *GCLC* was unaffected by DTT (Figure 2A,B). Conversely, such DTT-induced expression of *GCLM* was completely abolished in *caNrf2*^Δ*N*^ cells, although its basal expression was enhanced, while significantly inducible expression of *GCLC* was greatly lagged until 20 h to 24 h of treatment. Collectively, these imply that differential expression of *GCLM* and *GCLC* is monitored by inter-regulated Nrf1 and Nrf2 in a time-dependent fashion. 

Taking *WT* (at *t*_0_) as the control, basal protein abundances of GCLC and GCLM were upregulated in *Nrf1α^−/−^* and *caNrf2*^Δ*N*^ cell lines, but significantly down-regulated in *Nrf2^−/−^*^Δ*TA*^ cells (Figure 2(c1,c2)). However, no significant changes or even decreases in DTT-inducible GCLC and GCLM proteins for shorter terms of 1 h to 2 h were determined in all examined cell lines (Figure 2C and Figure S2). Upon DTT intervention of *WT* cells for the longer durations, significant changes in GCLC and GCLM proteins were observed to increment with the increasing time of administration (Figure 2(d1,d2)). After knockout of *Nrf1α^−/−^*, GCLM changed rather significantly compared to GCLC, with a time-dependent gradual increase (Figure 2(e1,e2)); knockout of *Nrf2^−/−^*^Δ*TA*^ caused *GCLM* expression to become fainter, while GCLC appeared to be unaltered in comparison to the *WT* (at *t*_4*h*_) control (Figure 2(f1,f2)). Additionally, *caNrf2*^Δ*N*^ cells had no further stimulated increases in GCLC and GCLM in response to DTT, although their basal levels were higher than that of the *WT* (at *t*_4*h*_) (Figure 2(g1,g2)). 

As mentioned herein, the oxidized glutathione disulfide (GSSG) can be reduced to GSH form by glutathione-disulfide reductase (GSR, a central enzyme of antioxidant defense), in this redox cycle, where glutathione peroxidase 1 (GPX1) can also achieve the purpose of oxidative detoxification in cells by reducing some peroxides. When compared with *WT* (at *t*_0_) cells, basal mRNA levels of *GSR* and *GPX1* were evidently up-expressed in *Nrf1α^−/−^* and *caNrf2*^Δ*N*^ cell lines, but rather substantially down-expressed in *Nrf2^−/−^*^Δ*TA*^ cells (Figure 2H,I). After DTT treatment, transcriptional expression of *GSR* in *WT* cells was significantly up-regulated in a time-dependent manner from 12 h, whereas inducible expression of *GPX1* was modestly upregulated by this chemical. Intriguingly, a DTT-inducible decrease, rather than increase, of *GSR* occurred only with 12 h treatment of *Nrf1α^−/−^* cells (Figure 2H), as accompanied by obvious down-regulation of *GPX1* (Figure 2I). *Nrf2^−/−^*^Δ*TA*^ cells only displayed a lagged increase in mRNA expression of *GSR* at 24 h stimulation (Figure 2H), but biphasic induction of *GPX1* by DTT occurred respectively at 4 h and 20 h, although its basal levels were lowered (Figure 2I). Conversely, *caNrf2*^Δ*N*^ cells exhibited an obvious time-dependent DTT-inducible decrease in *GSR*, along with lagged induction of *GPX1* by this treatment for 20 h to 24 h, which was rather significantly higher than its basal levels (Figure 2I). Further examinations of GSR and GPX1 proteins revealed no significant changes in their inducible expression upon short-term intervention of all examined cells by DTT, although their basal levels were highly up-expressed in *Nrf1α^−/−^* and *caNrf2*^Δ*N*^ cell lines, but down-regulated in *Nrf2^−/−^*^Δ*TA*^ cells in comparison to the *WT* (at *t*_0_) control (Figure 2(c3,c4) and Figure S2). Continued detection of the longer-term DTT intervening effects on *WT* cells revealed almost no significantly inducible changes in GSR and GPX1 abundance, except from a marginal induction being lagged at 24 h (Figure 2(d3,d4)). Similarly to the control *WT* (at *t*_4_), almost no induction of GSR by DTT was observed in *Nrf1α^−/−^* cells, but as compared with enhanced expression of GPX1 in a time-dependent manner (Figure 2(e3,e4)). In *Nrf2^−/−^*^Δ*TA*^ cells, considerably fainter expression of GSR was not induced by DTT, while GPX1 was also not triggered by this drug (Figure 2(f3,f4)). However, *caNrf2*^Δ*N*^ cells displayed a modestly stimulated expression of GPX1, but not GSR in response to DTT (Figure 2(g3,g4)). Together, such distinct expression profiles of GSR and GPX1 at mRNA and protein levels may be attributable to coordinated inter-regulation by Nrf1 and Nrf2.

The expression of downstream genes *NQO1* and *HO-1* closely related to *Nrf1* and *Nrf2* was also increased with time-dependent induction of DTT in *WT* cells (Figure 2J,K). When compared with the *WT_t_*_0_ control, basal mRNA expression levels of *NQO1* and *HO-1* were upregulated in *Nrf1α^−/−^* cells, but down-regulated in *Nrf2^−/−^*^Δ*TA*^ cells, along with almost no changes in *caNrf2*^Δ*N*^ cells. The inducible expression of *HO-1* was further augmented by DTT stimulation of *Nrf1α^−/−^* cells for 12 h to 24 h (Figure 2K), whereas *NQO1* was largely unaffected by this chemical, except for a marginal increase lagged at 20 h (Figure 2J). Intriguingly, *NQO1* and *HO-1* were roughly unaltered or down-regulated by DTT, except for a slightly stimulated expression lagged at 24 h of this treatment in *Nrf2^−/−^*^Δ*TA*^ and *caNrf2*^Δ*N*^ cell lines. Further investigation of NQO1 and HO-1 revealed a largely similar trend of changes in their protein expression to that of mRNAs. In comparison to *WT* (at *t*_0_), basal NQO1 and HO1 expression abundances were enhanced in *Nrf1α^−/−^* cells, but suppressed in either *Nrf2^−/−^*^Δ*TA*^ or *caNrf2*^Δ*N*^ cells (Figure 2(c5,c6)). Short-term DTT intervention of examined cells for 1 h to 2 h also revealed no significant changes or even a modestly decreased trend in NQO1 and HO1 proteins. However, long-term intervention of *WT* cells with DTT from 4 h to 24 h led to gradually incremented abundances of NQO1 and HO-1 in a time-dependent fashion (Figure 2(d4,d6)). Further comparison with the *WT* (at *t*_4_) control indicated that DTT intervention of *Nrf1α^−/−^* cells for 4 h to 24 h also induced a gradual increased expression trend for NQO1, while HO-1 reached a relative inducible expression peak at 12 h and then gradually weakened (Figure 2(e5,e6)). In *Nrf2^−/−^*^Δ*TA*^ cells, NQO1 and HO-1 expression levels were not only weaker than those in *WT* (at *t*_4_), but also not induced by DTT (Figure 2(f5,f6)). Similarly, *caNrf2*^Δ*N*^ cells also manifested no significant changes in NQO1 and HO-1 in response to DTT, with the exception of only slight HO-1 induction at 24 h (Figure 2(g5,g6)).

Herein, we also examined the expression of *TALDO* (encoding a key enzyme in the non-oxidative pentose phosphate pathway to yield NADPH so as to maintain a reduced state of glutathione, thus protecting cells from oxygen free radicals [28]), *TKT* (encoding thiamine dependent enzyme to guide excess phosphate sugar to glycolysis in the pentose phosphate pathway), and *MT1E* and *MT2* (two members the metal sulfur family that act as antioxidants in the steady-state control of metals in cells and prevent the production of hydroxyl radicals [29]). As shown, biphasic changes in *MT1E* mRNA expression were observed in *WT* cells, which was first decreased, then gradually recovered and even increased as the time of DTT intervention was extended to 24 h of its maximum response. Loss of *Nrf1α^−/−^* led to a complete abolishment of basal and DTT-inducible mRNA expression of *MT1E* (Figure 2L). Intriguingly, similar results were obtained from *caNrf2*^Δ*N*^ cells. Conversely, loss of *Nrf2^−/−^*^Δ*TA*^ led to a substantial increase in basal *MT1E* expression, but its DTT-inducible expression changes were triphasic, which was first inhibited from 4 h to 12 h, then recovered at 16 h and induced to the maximum at 20 h, but finally returned to its basal level (Figure 2L). For *MT2*, only a lagged DTT-stimulated increase occurred at 24 h treatment of *WT* cells (Figure 2M). When compared with the *WT_t_*_0_ control, basal MT2 expression was significantly up-regulated in *Nrf1α^−/−^* and *caNrf2*^Δ*N*^ cell lines. A biphasic change in its DTT-inducible expression was exhibited in *Nrf1α^−/−^* cells. Similarly lagged DTT-triggering expression of MT2 was also observed in *caNrf2*^Δ*N*^ cells. Conversely, *Nrf2^−/−^*^Δ*TA*^ cells gave a marginal increase in basal MT2 levels; its DTT-stimulated expression was completely abolished (Figure 2M). Further examination revealed that basal mRNA expression levels of *TALDO* and *TKT* were up-regulated in *Nrf1α^−/−^* and *caNrf2*^Δ*N*^, rather than *Nrf2^−/−^*^Δ*TA*^, cell lines, when compared with the *WT_t0_* control (Figure 2N,O). Similar changes in *TALDO* protein levels were obtained (Figure 2C). Interestingly, significant increases in *TALDO* and *TKT* expression levels were stimulated by DTT in *WT* cells. Knockout of *Nrf1α^−/−^* or *Nrf2^−/−^*^Δ*TA*^ still gave rise to lagged induction of *TALDO* and *TKT* within 16 h to 24 h after DTT intervention (Figure 2N,O), while *caNrf2*^Δ*N*^ displayed a biphasic change in *TALDO and TKT* expression levels that was first decreased, and then recovered and increased during DTT stimulation with an inducible maximum occurring at 24 h (Figure 2N,O). However, little or no effect of DTT was observed on TALDO protein expression in all examined cells (Figure 2C–G).

### 3.3. Differential Requirements of Nrf1 and Nrf2 for the ER Stress-Responsive Genes Stimulated by DTT

**Figure 3:**
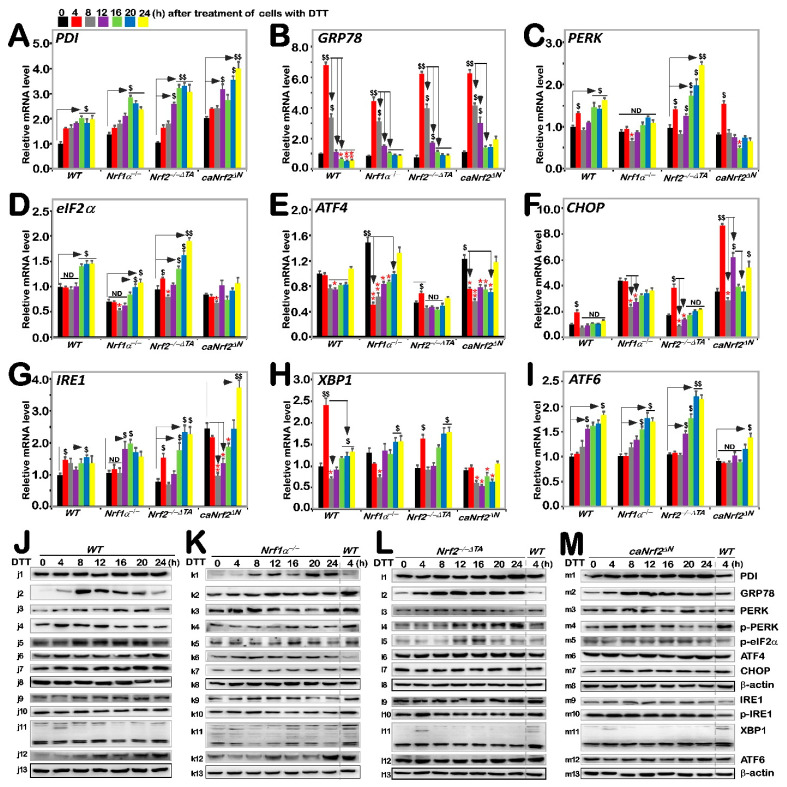
Distinct roles of Nrf1 and Nrf2 in the ER stress response induced by DTT. Four genotypic cell lines of *WT*, *Nrf1α^−/−^*, *Nrf2^−/−^*^△^*^TA^* and *caNrf2*^△^*^N^* were or were not treated with 1 mM DTT for 0 to 24 h, before basal and DTT-inducible mRNA expression levels of those ER stress-related genes were determined by RT-qPCR. Those genes included PDI (**A**), GRP78 (**B**), PERK (**C**), eIF2α (**D**), ATF4 (**E**), CHOP (**F**), IRE1 (**G**), XBP1 (**H**) and ATF6 (**I**). The data were shown as fold changes (*mean* ± *SD*, *n* = *3* × *3*), which are representative of at least three independent experiments being each performed in triplicate. Significant increases (*$*, *p* < *0.05; $$*, *p* < *0.01*) and significant decreases (* p < 0.05; ** p < *0.01*), in addition to the non-significance (ND), were statistically analyzed when compared with their corresponding controls (measured at 0 h). The main tendencies of up- and down- regulatory changes of different genes after 1 mM DTT intervention are also shown in Figure S5B. After similar treatment of *WT* (**J**), *Nrf1α^−/−^* (**K**), *Nrf2^−/−^*^△^*^TA^* (**L**), and *caNrf2*^△^*^N^* (**M**) cell lines for distinct lengths of time (i.e., 0, 4, 8, 12, 16, 20, 24 h), those inducible protein changes of PDI (**j1**–**m1**), GRP78 (**j2**–**m2**), PERK (**j3**–**m3**), p-PERK (**j4**–**m4**), p-eIF2α (**j5**–**m5**), ATF4 (**j6**–**m6**), CHOP (**j7**–**m7**), *β*-actin (**j8**–**m8**), IRE1 (**j9**–**m9**), p-IRE1 (**j10**–**m10**), XBP1 (**j11**–**m11**), ATF6 (**j12**–**m12**) and *β*-actin (**j13**–**m13**) were determined by Western blotting with indicated antibodies, whilst *β*-actin served as a loading control. The intensity of those immunoblots, representing different protein expression levels, was also quantified by the Quantity One 4.6.2 software, as shown in Figure S3.

It was reported that Nrf2 is significantly up-regulated by aggregated β-amyloid-mediated ER stress and activated by PERK as a canonical ER stress sensor [30,31]. Our previous work had shown differential and integral roles of Nrf1 and Nrf2 in mediating the unfolded protein response (i.e., UPR^ER^) to the classic ER stressor tunicamycin [20]. Herein, we examined whether Nrf1 and Nrf2 are required for monitoring putative ER stress response to DTT, which interferes with disulfide bond formation during protein folding towards maturation. Thus, it was reasoned that one of the first targets of oxidative protein folding attacked by DTT should be protein disulfide isomerase (PDI, which can also act as a reductase to cut those protein disulfide bonds attached to the cell surface, aside from an ER chaperone to inhibit misfolded protein aggregation [32,33]). As expected, DTT-stimulated mRNA expression levels of *PDI* were up-regulated in all four examined cell lines, even upon loss of *Nrf1α^−/−^* or *Nrf2^−/−^*^Δ*TA*^, but its basal enhancement occurred in *Nrf1α^−/−^* and *caNrf2*^Δ*N*^, rather than *Nrf2^−/−^*^Δ*TA*^, cell lines (Figure 3A). Such a rebound effect on transcriptional expression of *PDI* is likely controlled by a feedback loop coordinated with Nrf1 and Nrf2. However, its protein expression abundances were significantly incremented by DTT in *Nrf1α^−/−^* cells, but only modestly enhanced in the other three cell lines (Figure 3J–M). This implies the possibly enhanced stability of PDI may be attributable to *Nrf1α^−/−^*-impaired proteasomal degradation, particularly under reductive stress conditions.

Next, to our surprise, we found that mRNA expression levels of GRP78 (a pivotal partner with three ER sensors, PERK, IRE1 and ATF6) were rapidly increased to its maximum induction by 4 h intervention of DTT in all four examined cell lines, and then gradually decreased to its basal levels or to rather lower extents, as stimulation time was extended to 24 h (Figure 3B). Similar biphasic changes in GRP78 protein abundance were also determined in these four cell lines (Figure 3(j2–m2)). However, it is notable that the extents of DTT-inducible mRNA and protein expression in *Nrf1α^−/−^* cells were rather lower than those obtained from *WT*, *Nrf2^−/−^*^Δ*TA*^ and *caNrf2*^Δ*N*^, cell lines, but almost no differences in induction of GRP78 were observed between *Nrf2^−/−^*^Δ*TA*^ and *caNrf2*^Δ*N*^ cell lines. This indicates that Nrf1, rather than Nrf2, is required for bidirectional regulation of GRP78 at distinct levels in response to DTT. 

By further examination of three ER stress responsive genes, it was revealed that a modest bimodal induction of *PERK* by DTT occurred early at 4 h and later after 16 h of this chemical treatment, respectively, in *WT* cells, and such bimodality was further augmented in *Nrf2^−/−^*^Δ*TA*^ cells (Figure 3C), but substantially blunted in *Nrf1α^−/−^* cells. Of note, the early peak was abolished by knockout of *Nrf1α*, while the latter peak was abrogated and even suppressed by *caNrf2*^Δ*N*^. This implies that Nrf1 and Nrf2 bi-directionally positively and negatively regulate expression of *PERK*, particularly its lagged induction by DTT, respectively. In *WT* cells, phosphorylated protein of p-PERK was significantly stimulated by DTT at 4 h and then decreased to rather lower extents (Figure 3(j4)). A similar, but modest, induction pattern of p-PERK by DTT was manifested in *caNrf2*^Δ*N*^ cells (Figure 3(m4)), but this induction seemed to be abolished by knockout of *Nrf1α^−/−^* (Figure 3(k4)). Conversely, in *Nrf2^−/−^*^Δ*TA*^ cells, DTT-inducible expression of p-PERK was gradually incremented from 12 h to 24 h of its maximum (Figure 3(l4)). In addition, no significant changes in total PERK were observed in all four examined cell lines, except for partial attenuation by knockout of *Nrf1α^−/−^* within an indicated period of time (Figure 3J–M and Figure S3). Collectively, induction of the PERK signaling by DTT is also positively and negatively monitored by Nrf1 and Nrf2, respectively. 

The downstream eIF2α-ATF4-CHOP of PERK signaling was also examined herein. The results showed only marginal induction of *eIF2α* and *CHOP* at mRNA expression levels after 16 h or early at 4 h of DTT intervention, respectively, along with no induction or even decreases in *ATF4* in *WT* cells (Figure 3D–F). By contrast, basal and/or DTT-inducible *eIF2α* expression levels were repressed by *Nrf1α^−/−^*, but enhanced by *Nrf2^−/−^*^Δ*TA*^, even though unaffected by *caNrf2*^Δ*N*^ (Figure 3D). In addition, the phosphorylated eIF2α expression was modestly induced by DTT in *Nrf2^−/−^*^Δ*TA*^ cells, but no marked changes were observed in the other three cell lines (Figure 3J–M and Figure S3). These indicate that Nrf1 and Nrf2 regulate eIF2α in a way similar to their monitoring of PERK expression. However, DTT intervention led to significant down-regulation of *ATF4* in *Nrf1α^−/−^* and *caNrf2*^Δ*N*^ cell lines when compared with *WT* cells, although upregulation of its basal mRNA expression levels in *Nrf1α^−/−^* and *caNrf2*^Δ*N*^ cells occurred with their suppression in *Nrf2^−/−^*^Δ*TA*^ cells (Figure 3E). However, no striking changes in ATF4 protein levels were observed in all examined cells (Figure 3J–M). Hence, it is inferable that Nrf2 may be required for DTT-stimulated trans-repression of *ATF4*. Moreover, only early induction of *CHOP* by DTT occurred, to a lower degree, at 4 h treatment of *WT* cells and was further amplified in *Nrf2^−/−^*^Δ*TA*^ and *caNrf2*^Δ*N*^, but not in *Nrf1α^−/−^* cell lines, although its basal expression was also upregulated in *Nrf1α^−/−^* cells (Figure 3F). Additionally, no significant changes in CHOP protein levels were determined in all four cell lines (Figure 3J–M). This implies that Nrf1 is likely required for early induction of *CHOP* by DTT. Overall, these findings indicate differential and integral roles of Nrf1 and Nrf2 in monitoring the PERK signaling to its downstream eIF2α-ATF4-CHOP pathway. 

A bimodal of the IRE1-XBP1 signaling at their mRNA levels was also induced by DTT in *WT* cells. Such induction of *IRE1* was significantly enhanced in *Nrf2^−/−^*^Δ*TA*^ cells, though its basal levels were down-regulated (Figure 3G). The early peak of *IRE1* induction by DTT was abrogated or suppressed in *Nrf1α^−/−^* or *caNrf2*^Δ*N*^ cells, but its later peak was unaffected or augmented in the two cell lines, respectively. The marked early peak of *XBP1* induced by DTT was observed in *WT* cells, decreased by *Nrf2^−/−^*^Δ*TA*^ and abolished by *Nrf1α^−/−^* or *caNrf2*^Δ*N*^ (Figure 3H), whereas the secondary peak was also abolished by *caNrf2*^Δ*N*^, but unaffected by *Nrf1α^−/−^* *or Nrf2^−/−^*^Δ*TA*^. Furthermore, time-dependent increments of *ATF6* mRNA expression occurred from 12 h to 24 h in DTT-treated *WT*, *Nrf1α^−/−^* or *Nrf2^−/−^*^Δ*TA*^, but not *caNrf2*^Δ*N*^ cells (Figure 3I). In addition, no obvious changes in IRE1, p-IRE1 and XBP1 proteins were observed in all four examined cell lines, but ATF6 protein levels were, to different extents, enhanced by DTT in *WT*, *Nrf1α^−/−^*, *caNrf2*^Δ*N*^, rather than *Nrf2^−/−^*^Δ*TA*^, cell lines (Figure 3J–M). Taken together, these findings demonstrate that Nrf1 and Nrf2 differentially regulate the ER-stress responsive genes to DTT. 

### 3.4. Distinct Intracellular Redox Changes among Different Genotypic Cell Lines in Response to DTT

**Figure 4:**
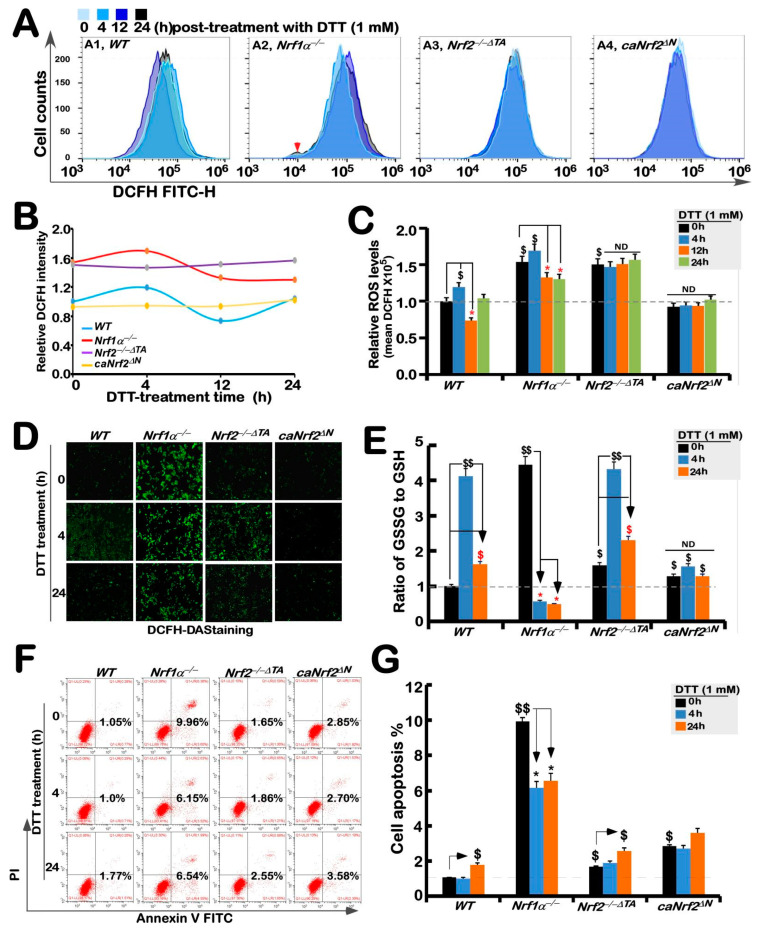
Changes in both ROS levels and ratio of GSSG to GSH are accompanied by distinct apoptosis of different genotypic cell lines against DTT. (**A**) Experimental cell lines *WT*, *Nrf1α^−/−^*, *Nrf2^−/−^*^△^*^TA^* and *caNrf2*^△^*^N^* were treated with 1 mM DTT for different time periods (i.e., 0, 4, 12 and 24 h), before they were subjected to flow cytometry analysis of their intracellular ROS levels by the DCFH-DA fluorescence intensity. The data for ROS products and differences were further analyzed by FlowJo 7.6.1 software, as shown in (**B**,**C**). (**D**) After DCFH-DA staining, the ROS fluorescent images in different cell lines were obtained under microscope. (**E**) Measurements of the intracellular GSH and GSSH levels were performed and also repeated three times, each of which was performed in triplicate. Statistical significances were calculated: *$$*, *p* < *0.01*, and *$*, *p* < *0.05* indicate significant increases by comparing each basal value of [*Nrf1α^−/−^*]*_T_*_0_, [*Nrf2^−/−^*^△^*^TA^*]*_T_*_0_, and [*caNrf2*^Δ*N*^]*_T_*_0_ with that of [*WT*]*_T_*_0_, while both * p < *0.05* denote significant decreases in those values from each of the cell lines treated by DTT for 4 h (i.e., [X]*_T_*_4_) or 24 h (i.e., [X]*_T_*_24_) versus its untreated [X]*_T_*_0_ value in the same groups. (**F**) Distinct cell lines of *WT*, *Nrf1α^−/−^*, *Nrf2^−/−^*^△^*^TA^* and *caNrf2*^△^*^N^* were (or were not) treated with 1 mM DTT for different lengths of time. Subsequently, the cells were incubated with a binding buffer containing Annexin V-FITC and propidium iodide (PI) for 15 min, before being subjected to the flow cytometry analysis of apoptosis. (**G**) The final results were shown by the column charts, which were representative of at least three independent experiments, each performed in triplicate. Of note, *$$*, *p* < *0.01*, and *$*, *p* < *0.05* indicate significant increases in each basal value of [*Nrf1α^−/−^*]*_T_*_0_, [*Nrf2^−/−^*^△^*^TA^*]*_T_*_0_, and [*caNrf2*^Δ*N*^]*_T_*_0_ with that of [*WT*]*_T_*_0_, while both * p < *0.05* denote significant decreases in those values from each of the cell lines treated by DTT for 4 h (i.e., [X]*_T_*_4_) or 24 h (i.e., [X]*_T_*_24_) versus the untreated [X]*_T_*_0_ value in the same groups.

As a collective term, ROS include superoxide anion, hydrogen peroxide, and all other oxygenated active substances, but are still hard to be accurately detected, because they have strong oxidation activity to be exerted for a short retention time while they are easy to be removed by antioxidants. Therefore, visualization of ROS by fluorescence microscopy and its quantification by flow cytometry are usually used to detect their changing status evaluated by the green fluorescence raised from the DCFH dye reaction with intracellular ROS, to assess the difference in antioxidant capacity [34]. The results showed that basal ROS levels in *Nrf1α^−/−^* cells were obviously higher than that of *WT_t_*_0_, while a slight increase in the yield of ROS was observed after 4 h stimulation by DTT, before being decreased to lower extents than its basal levels (Figure 4A–C). Such a slight DTT-stimulated rise in ROS was also observed in *WT* cells before being decreased and then recovered to its basal levels. By contrast, basal ROS levels were also apparently increased in *Nrf2^−/−^*^Δ*TA*^ cells, but this status seemed to be unaffected by DTT intervention (Figure 4A–C). Intriguingly, no differences in basal and DTT-stimulated ROS levels in *caNrf2*^Δ*N*^ cells were determined by comparison to *WT* controls. Besides, similar changes in the intensity of fluorescence arising from intracellular DCFH-DA dye observed by microscopy were also fully consistent with the results as described above (Figure 4D). Altogether, these findings demonstrate that although both Nrf1 and Nrf2 are responsible for endogenous antioxidant cytoprotection, Nrf1 rather than Nrf2 is required for DTT-stimulated antioxidant defense response. 

Further experiments revealed that an intrinsic significant augmentation in the proportion of GSSG to GSH was de facto in *Nrf1α^−/−^* cells, whereas *Nrf2^−/−^*^Δ*TA*^ cells only had a modestly enhanced ratio of GSSG to GSH, when compared with the *WT_t_*_0_ control (Figure 4E). Upon stimulation of DTT for 4 h, an inducible elevated rate of GSSG to GSH was examined only in *WT* and *Nrf2^−/−^*^Δ*TA*^, but not *Nrf1α^−/−^* *or caNrf2*^Δ*N*^ cells, which seemed to be the highly endogenous level obtained from *Nrf1α^−/−^* cells. When stimulation with DTT extended to 24 h, such DTT-stimulated elevation descended closely to basal levels (Figure 4E). Notably, DTT intervention of *Nrf1α^−/−^* cells for 4 h to 24 h resulted in substantially decreased rates of GSSG to GSH, but no changes in the GSSG to GSH ratio were observed in *caNrf2*^Δ*N*^ cells. From these, it is inferable to be attributable to aberrant accumulation of hyperactive Nrf2 in *Nrf1α^−/−^* cells insomuch as to reinforce its antioxidant and detoxifying cytoprotection against DTT, whereas *caNrf2*^Δ*N*^ cells with a genetic deletion of the *N*-terminal Keap1-binding domain lost their powerful response to the redox-sensing by Keap1. 

Further experimental evidence was also obtained from flow cytometry analysis of cell apoptosis, revealing the lowest apoptosis level among untreated *WT* cells, while a relatively higher number of *Nrf1α^−/−^* cells underwent apoptosis (Figure 4F,G). Almost similar apoptosis of *Nrf2^−/−^*^Δ*TA*^ and *caNrf2*^Δ*N*^ cells was close to that of *WT* cells, but much lower than that of *Nrf1α^−/−^* cells (Figure 4F). However, DTT stimulation caused significant decreases in apoptosis of *Nrf1α^−/−^* cells, but slightly increased apoptosis of *Nrf2^−/−^*^Δ*TA*^ or *caNrf2*^Δ*N*^ cells at 24 h after this chemical treatment, along with no changes in *caNrf2*^Δ*N*^ cells (Figure 4G). This demonstrates that accumulated Nrf2 in *Nrf1α^−/−^* cells still exerted its intrinsic cytoprotective effect against DTT-induced apoptosis, but this effect was lost in both *Nrf2^−/−^*^Δ*TA*^ and *caNrf2*^Δ*N*^ cell lines (the former *Nrf2^−/−^*^Δ*TA*^ lacks its transactivation domain to regulate its target genes, while the latter *caNrf2*^Δ*N*^ lacks its responsive interaction with the redox-sensing Keap1).

### 3.5. The Redox Status of Cys342 and Cys640 in Nrf1 Is Required for Their Protein Stability and Trans-Activity

**Figure 5:**
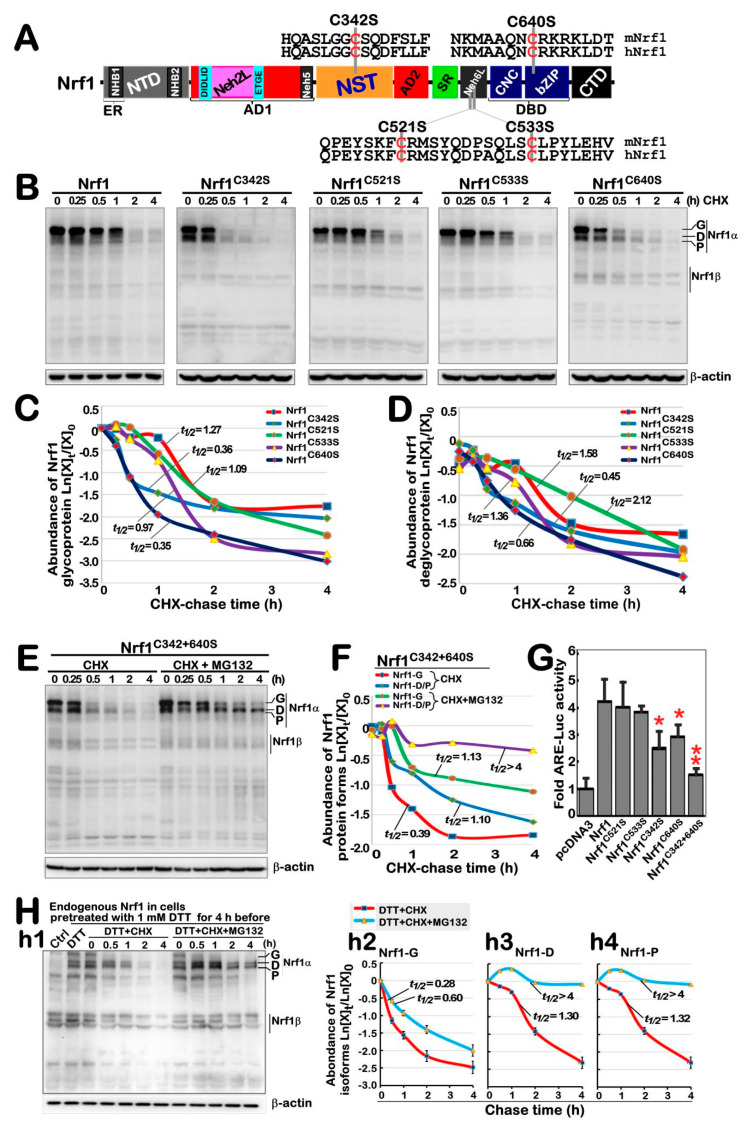
Nrf1 stability and its *trans*-activity were determined by redox status of its Cys342 and Cys640. (**A**) Schematic diagram of four cysteine mutants within Nrf1, which are distributed in its NST, Neh6L and bZIP domains. (**B**) Stability of Nrf1 and its mutants was determined by the pulse-chase experiments for 0 to 4 h intervention by CHX (50 μg/mL). (**C**,**D**) The results were evaluated for the turnover trend of glycoproteins (**C**) and deglycoproteins (including processed proteins) (**D**) of Nrf1α and mutants, with distinct half-lives estimated. (**E**,**F**) After treatment of cells with CHX alone or plus 10 μM MG132, stability of the Nrf1^C342/640S^ mutant was further examined by pulse-chase experiments (**E**), and distinct half-lives of its derivative proteins were calculated (**F**). (**G**) *WT* cells were co-transfected with an *ARE-Luc* reporter, together with each of the indicated expression constructs for Nrf1, its mutants or empty pcDNA3.1 vector. The luciferase activity was normalized to their internal controls and corresponding backgrounds obtained from the co-transfection of cells with non-*ARE* reporter and each of the expression constructs. The results were calculated as *mean* ± *SD* (*n* = *6* × *3*) (* p < 0.05; ** p < *0.01*) relative to the basal activity (at a given value of 1.0) obtained from the transfection of cells with empty pcDNA3.1 vector and ARE-driven reporter. (**H**) Stability of endogenous Nrf1α proteins was examined by pulse-chase experiments after stimulation of cells by 1 mM DTT (**h1**), and changes in its derived glycoprotein, deglycoprotein, and processed protein abundances were represented by their respective turnover time-course curves (**h2**–**h4**). In addition, similar examinations of ectopically expressing Nrf1α and its Nrf1^C342/640S^ mutant after 1 mM DTT stimulation were also shown in Figure S4. The intensity of relevant immunoblots representing different protein expression levels was also quantified by the Quantity One 4.6.2 software. The resulting data were shown graphically, after being calculated by a formula of Ln ([X]_t_/[X]_0_), in which [X]_t_ indicated a fold change (*mean* ± *SD*) in each of those examined protein expression levels at different times relative to corresponding controls measured at 0 h (i.e., [A]_0_), which were representative of at least three independent experiments.

Our previous studies showed that Nrf1 undergoes a variety of post-translational modifications, such as glycosylation, deglycosylation, ubiquitination and phosphorylation, as well as selectively proteolytic processing [13]. Here, we investigated which cysteine (Cys) residues within Nrf1 can directly sense the reductive stressor DTT (that can reduce protein disulfide bond (-*S*-*S*-) to sulfhydryl (-*SH*) and also oxidize itself into six-membered ring, to assist in maintaining protein function [35]). A schematic shows mutagenesis mapping of four Cys residues of Nrf1 into serines (i.e., C342S, C521S, C533S and C640S) within its NST, Neh6L and bZIP domains, respectively (Figure 5A). Next, the pulse-chase experiments revealed that a considerable portion of the full-length glycoproteins of two mutants, Nrf1^C342S^ and Nrf1^C640S^, were rapidly converted into their deglycoproteins and then proteolytically processed to disappear in a faster manner than those equivalents arising from wild-type Nrf1 (Figure 5B). Protein stability was evaluated by Western blotting to measure the half-lives of Nrf1^C342S^, Nrf1^C640S^ and wild-type Nrf1, which were calculated for their glycoprotein turnover to be 0.36 h (21.6 min), 0.35 h (21 min) and 1.27 h (76.2 min) (Figure 5C), and for their deglycoprotein turnover to be 0.45 h (27 min), 0.66 h (39.6 min) and 1.58 h (94.8 min) (Figure 5D), respectively, after treatment with CHX (to inhibit the nascent polypeptide synthesis). By contrast, the stability of another two mutants Nrf1^C521S^ and Nrf1^C533S^ were only marginally affected, when compared with that of wild-type Nrf1 (Figure 5B–D). 

Further examination of a bi-Cys mutant Nrf1^C342/640S^ revealed that the half-lives of its glycoprotein and deglycoprotein turnover were calculated to be 0.39 h (23.4 min) and 1.10 h (66.0 min), respectively, after CHX treatment (Figure 5E,F). Upon addition of proteasomal inhibitor MG132 plus CHX, the half-life of this mutant glycoprotein was only modestly extended to 1.13 h (67.8 min), just because it had to be deglycosylated during the pulse-chase experiments. Rather, the half-life of this mutant deglycoprotein was significantly extended to over 4 h before stopping this experiment (Figure 5E,F). This implies that this deglycoprotein turnover is quality-controlled by proteasome-mediated degradation pathway. Interestingly, transactivation activity of *ARE*-driven luciferase reporter gene regulated by Nrf1 was significantly inhibited by its mutants Nrf1^C342S^, Nrf1^C640S^ or Nrf1^C342/640S^ (Figure 5G). Therefore, it is inferable that the redox state of both Cys342 and Cys640 residues in Nrf1 could be required for its protein stability and transcriptional activity.

Next, the effect of DTT-induced redox stress on Nrf1 stability was further determined. The results showed that DTT enhanced accumulation of all the endogenous Nrf1-drived proteins, and they were further accumulated by MG132 plus DTT (Figure 5(h1)). However, the half-lives of Nrf1 glycoprotein (i.e., Nrf1-G), deglycoprotein (i.e., Nrf1-D) and its processed protein (i.e., Nrf1-P) were measured to be 0.28 h (16.8 min), 1.30 h (78 min), and 1.32 h (79.2 min), respectively, after DTT co-treatment of cells with CHX (Figure 5(h2–h4)). Upon addition of MG132 to DTT/CHX-treated cells, the half-life of Nrf1-G was slightly extended to 0.60 h (36 min), whereas the half-lives of Nrf1-D and Nrf1-P were strikingly prolonged to over 4 h after stopping experiments. Further determination of Nrf1 protein turnover was carried out by Western blotting of ectopically expressed isoforms of this CNC-bZIP factor and its bi-Cys mutant Nrf1^C342/640S^, which were resolved by gradient LDS-NuPAGE gels containing 4–12% polyacrylamides (Figure S4). The results revealed that abundance of Nrf1 was enhanced by DTT (Figure S4A). In the presence of DTT, the half-lives of Nrf1-G, -D and -P were calculated to be 0.18 h (10.8 min), 1.06 h (63.6 min) and 0.74 h (44.4 min), respectively, after CHX treatment (Figure S4(a2–a4)). By contrast, Nrf1^C342/640S^ appeared to be unaffected by this chemical (Figure S4(b1)), with distinct half-lives of its isoforms that were slightly changed to be 0.24 h (14.4 min), 0.64 h (38.4 min) and 0.57 h (34.2 min), respectively after co-treatment of DTT with CHX (Figure S4(b2–b4)). Notably, the glycoprotein half-life of Nrf1 or Nrf1^C342/640S^ was only modestly extended by addition of MG132, to 0.30 h (18 min) or 0.50 h (30 min), respectively, but their deglycoproteins and proteolytic proteins were all significantly prolonged to over 4 h. Taken together, these data indicate that the redox state of Cys342 and Cys640 residues in Nrf1 is not only required for its protein stability, but that both may also serve as a redox sensor for the DTT stressor.

### 3.6. Biphasic Effects of DTT on Transcriptional Expression of Nrf1-Target Proteasomal Genes

**Figure 6:**
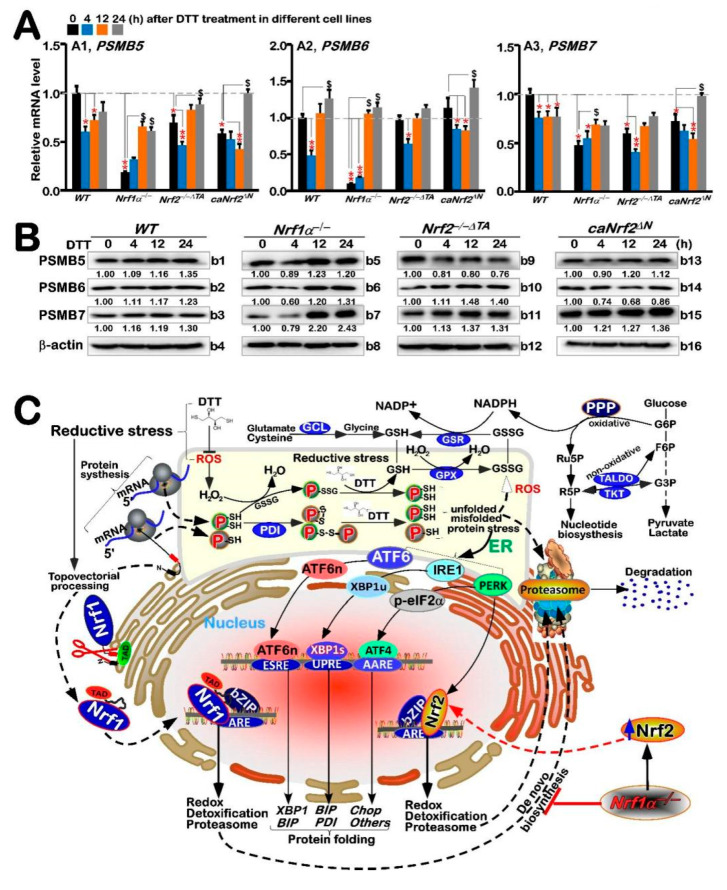
Differential expression of proteasomal genes regulated by Nrf1-independent pathway under reductive stress induced by DTT. (**A**) Four genotypic cell lines of *WT*, *Nrf1α^−/−^*, *Nrf2^−/−^*^△^*^TA^* and *caNrf2*^△^*^N^* were (or were not) treated with 1 mM DTT for 0 to 24 h, before both basal and DTT-inducible mRNA expression levels of core proteasomal subunit genes (i.e., *PSMB5* (**A1**), *PSMB6* (**A2**) and *PSMB7* (**A3**)) were determined by RT-qPCR. The resulting data were shown as fold changes (*mean* ± *SD*, *n* = *3* × *3*), which are representative of at least three independent experiments, each performed in triplicate. Significant increases (*$*, *p* < *0.05*) and significant decreases (* p < *0.05*; ** p < *0.01*) were statistically calculated by comparison with their corresponding controls (measured at 0 h). (**B**) These proteasomal protein expression levels in four distinct cell lines were determined by Western blotting with their indicated antibodies, such *PSMB5* (**b1**, **b5**, **b9**, **b13**), *PSMB6* (**b2**, **b6**, **b10**, **b14**) and *PSMB 7*(**b3**, **b7**, **b11**, **b15**), whilst *β*-actin served as a loading control. The intensity of those immunoblots, representing different protein expression levels, was also quantified by the Quantity One 4.6.2 software, as shown on *bottom* (**C**). A model was proposed to provide a better explanation of distinct roles for Nrf1 and Nrf2 in DTT-stimulated reductive stress response, along with ER stress signaling and relevant redox metabolism.

It was previously reported that Nrf1, rather than Nrf2, plays an essential role in controlling transcriptional expression of all proteasomal subunit genes [36]. Such expression of the proteasomal genes regulated by Nrf1 is required for the ER-associated degradation (ERAD), as accompanied by induction of three classical response pathways driven by the ER stress-sensing genes (i.e., *PERK*, *IRE1* and *ATF6*). Therefore, we explored whether DTT-stimulated Nrf1 and Nrf2 are also required for the expression of key proteasomal (*PSM*) genes along with ER signaling networks. The RT-qPCR results showed significant decreases in basal mRNA expression levels of all three examined genes *PSMB5*, *PSMB6* and *PSMB7* in *Nrf1α^−/−^* cells (Figure 6A), while basal *PSMB5* and *PSMB7* expression levels were also partially decreased in *Nrf2^−/−^*^Δ*TA*^ or *caNrf2*^Δ*N*^ cells, when compared with *WT_t0_* controls. This implies that except for Nrf1, Nrf2 is also partially involved in regulating transcription of some PSM genes (e.g., *PSMB5* and *PSMB7*) through its *N*-terminal Keap1-binding domain, in addition to its transactivation domain. 

Interestingly, DTT stimulation of *Nrf1α^−/−^* cells (with accumulation of hyperactive Nrf2) caused evident increases in *PSMB5*, *PSMB6* and *PSMB7* at mRNA and protein expression levels close to basal *WT_t0_* controls (Figure 6A or Figure 6B,(b5–b7)). This indicates such up-regulation of proteasomal genes by DTT may occur through Nrf1-independent and/or Nrf2-dependent pathways. In *WT* cells, DTT caused partial decreases in *PSMB5* and *PSMB7*, and also biphasic changes (i.e., decreased early, then recovered and even elevated) in *PSMB6* (Figure 6A), but their protein abundances were almost unaltered (Figure 6(b1–b3)). Similarly, DTT-inducible biphasic expression of all three examined genes was also observed in *Nrf2^−/−^*^Δ*TA*^ or *caNrf2*^Δ*N*^ cell lines, with no obvious changes in their protein levels. Together, these findings suggest transcriptional expression of proteasomal genes is also tightly regulated by other not-yet-identified factors (e.g., Bach1), along with Nrf1 and Nrf2, particularly under DTT-stimulated stress conditions, although the basal proteasomal expression is predominantly governed by Nrf1, as well partially by Nrf2. 

## 4. Discussion 

The concept of ‘stress’, as first put forward by Selye H, a Canadian physiologist, represents a state of tension when an individual feels threatened physically and mentally [37]. A similar concept of stress is also employed in cell biology, and the relevant topic has become an attractive direction to be followed by a large number of researchers from different fields. Most studies on animal stress are primarily focused on cell physiological factors, so mechanistic studies of cell stress have provided part of the theoretical basis for animal stress. The occurrence of cell stress enhances the ability of cells to resist damage and thus survive under adverse conditions. Such results are caused by distinct stressors, but in short, oxidative stress is still widely concerned in life science, medical and other related fields. Interestingly, there is a close relationship between oxidative stress and ER stress, because oxidative stress induced by pathological stimuli can also provoke the occurrence of ER stress, and vice versa. This implies there exists a mutually promoting positive feedback circuit between both oxidative and ER stresses [38]. 

As a matter of fact, the ER lumen is known as the most oxidizing subcellular compartment in cells, which facilitates formation of disulfide bonds in relevant protein structures and their proper folding. This process is also accompanied by ROS, to be released under ER stress, although the mitochondria are viewed as a key site for ROS byproducts. Such oxidative circumstances are provided for synthesis of biological macromolecules in the ER. In the meantime, appropriate oxidative stress triggers a certain hormetic effect to assist in the reprocessing of unfolded and misfolded proteins in the ER. Rather, the excessive ROS cause ER stress, resulting in accumulation of unfolded and misfolded proteins. Such UPR effect can also be triggered by the functional load of ER. Under normal conditions, the ER stress sensors IRE1, PERK, and ATF6 bind to GRP78 in the ER and also remain inactive. Dissociation of GRP78 from the ER lumen leads to the oligomerization of transmembrane protein IRE1, transfer of ATF6 to the Golgi apparatus, and activation of PERK, respectively (Figure 6C). The latter PERK phosphorylates and activates eIF2α so as to terminate the general translation process, thus reducing the internal load of ER. The active phosphorylated IRE1 splices downstream *XBP1* mRNA to give rise to the transcriptional activity of XBP1s (i.e., spliced *XBP1* encodes a longer polypeptide than its un-spliced prototype *XBP1u* (with resistance to *XBP1s’* function), which enters the nucleus and continuously activates UPR genes by binding the ER stress elements (ERSEs) in their promotor regions [39,40]. These indicate a complex and large regulatory relationship network between the UPR and ER stress and the occurrence of oxidative stress. In UPR response, CHOP acts as a pro-apoptotic factor to mediate the activation of ER stress-related apoptotic pathways. To combat overproduction of ROS, almost all cellular life forms have evolutionarily been equipped with an array of naturally powerful antioxidant defenses. These include a series of essential antioxidant, detoxification and cytoprotective mechanisms controlled by the CNC-bZIP transcription factors [41]. In this family, Nrf1 can activate transcriptional expression of Herpud1 (homocysteine-inducible ER-resident protein with ubiquitin-like domain 1, also called Herp1, that is involved in both UPR and ERAD) driven by its ARE battery, and the resulting ubiquitin complex is transferred to the proteasome in order to promote the unfolded and misfolded protein degradation, thereby preventing or alleviating the ER-overloaded stress [42]. This notion is supported by experimental evidence that endogenous ER stress signaling to activate UPR occurs in steatotic hepatocytes with homozygous knockout of *Nrf1^−/−^*, but not of *Nrf2^−/−^* [23]. Therefore, it is inferable that Nrf1 plays an important role in maintaining the ER redox homeostasis, particularly upon sensing intracellular protein, lipid, glucose and redox challenges. The loss of this function in mice results in overtly severe pathological consequences that are caused through *Nrf1α^−/−^* cell proliferation and malignant transformation, leading to spontaneous development of non-alcoholic steatohepatitis and hepatocellular carcinoma [43]. 

In striking contrast to oxidative stress, only a small number of 7864 entities about reductive stress were searched from the PubMed database until 24 May 2022. In this study, we examined distinct responsive effects of Nrf1 and Nrf2 to DTT-stimulated reductive stress. This was due to the inherent ability of DTT to maintain the reduced state of protein sulfhydryl groups, which is crucial important for the stability of many protein functions. However, protein disulfide bond (-*S*-*S*-) was reduced by DTT into sulfhydryl (-*SH*), which affects proper folding of many proteins and even triggers ER stress induced by misfolded and unfolded proteins [44]. This is because the unstable cysteine (Cys) is the only amino acid with reducing sulfhydryl (-*SH*) in more than 20 amino acids that make up the proteins, and thus is prone to oxidation–reduction to be converted with the -S-S- bond of cystine. Herein, we found that the redox status of Cys342 (in Nrf1’s glycodomain) and Cys640 (in Nrf1’s DNA-binding domain) residues is required for this CNC-bZIP protein stability and *trans*-activity.

Nrf1 is a *de facto* mobile transmembrane protein with a dynamic membrane topology, which is somewhat similar to, but different from, that of PERK, IRE1 and ATF6 within and around the ER [20]. Herein, our evidence suggests that PERK, IRE1, and ATF6 are differentially activated along with differential expression of their cognate responsive genes by DTT treatment of different genotypic cell lines with the presence or absence of Nrf1 and Nrf2 (for different time-dependent effects). Specifically, the DTT-induced ERS response signaling pathway is accompanied by the transcriptional expression of Nrf1 and Nrf2. The bidirectional expression of PERK and eIF2α, which are positively and negatively regulated by Nrf1 and Nrf2, respectively, is accompanied by down-regulation of ATF4 by DTT, although the latter basal expression was upregulated by *Nrf1α^−/−^* and *caNrf2*^Δ*N*^. The IRE1-XBP1 and ATF6 were differentially up-regulated by DTT in *Nrf1α^−/−^* and *Nrf2^−/−^*^Δ*TA*^, but XBP1 appeared to be unaffected by *caNrf2*^Δ*N*^. These indicate that DTT-caused reductive stress leads to trans-repression of ATF4, but transactivation of XBP1 and ATF6, possibly dependent on Nrf1 and Nrf2; the latter *N*-terminal Keap1-binding domain is also required for this responsiveness to DTT. The UPR signaling-provoked chaperone GRP78 (also called BIP) was rapidly induced by DTT, and then was gradually decreased and even suppressed by this reductive stressor, whereas PDI (catalyzing disulfide bonds to be formed in proper folding proteins) was gradually upregulated by DTT as this treatment time was extended, in all examined cell lines, regardless of whether Nrf1 was present or Nrf2 lost either its *N*-terminal Keap1-binding domain or transactivation domain. This demonstrates not only that DTT can rapidly stimulate ER stress signaling and also provoke a rebound effect on PDI to increment its expression levels, but also that this process does not have to rely on Nrf1 or Nrf2, although both factors may be involved.

Upon stimulation of wild-type cells by DTT, the ARE-driven genes encoding GCLM, GCLC, HO-1, NQO1, GSR, TKT, and TALDO were up-regulated to different extents. Among them, basal and DTT-stimulated expression levels of GCLM, GCLC, HO-1 and TKT were further augmented in *Nrf1α^−/−^* cells, but obviously suppressed in *Nrf2^−/−^*^Δ*TA*^ cells, and almost unaffected in *caNrf2*^Δ*N*^ cells except from only lagged induction of GCLC. This implies they are Nrf2-dependent responsive genes to DTT, and are also regulated by its *N*-terminal Keap1-binding domain. By contrast, DTT-inducible expression of GSR, GPX1 and NQO1 were roughly unaffected by *Nrf1α^−/−^* and *Nrf2^−/−^*^Δ*TA*^, suggesting they are co-regulated by Nrf1 and Nrf2. Only marginally lagged stimulation of MT1E and MT2 by DTT was examined in wild-type cells, but totally abolished by knockout of *Nrf1α^−/−^* or *Nrf2^−/−^*^Δ*TA*^, respectively. Conversely, the formal MT1E’s basal and DTT-stimulated expression levels were significantly increased in *Nrf2^−/−^*^Δ*TA*^ cells, while the latter MT2 expression was modestly elevated in *Nrf1α^−/−^* cells, which occurred after 16 h treatment with DTT, but early treatment for 4 h to 12 h led to an overt inhibition. These findings indicate that MT1E is an Nrf1-specific target, whereas MT2 is another Nrf2-specific target, but both are not sensitive to DTT-leading reductive stress, particularly during early treatment, whereas the lagged induction may be triggered by other not-yet-unidentified mechanisms (in which an increase in ROS cannot be ruled out). Unexpectedly, a marginal increase in ROS levels was determined only for 4 h intervention of *WT* or *Nrf1α^−/−^* cell lines. The GSSG to GSH rate was increased by DTT in *WT* and *Nrf2^−/−^*^Δ*TA*^ cell lines, but repressed in *Nrf1α^−/−^* (with hyperactive Nrf2 accumulated) or almost unaffected by *caNrf2*^Δ*N*^. This suggests DTT may induce reductive stress through Nrf2 to yield a certain amount of GSH in *Nrf1α^−/−^* cells, which enables cytoprotection against this chemical cytotoxicity, whereas loss of *Nrf2^−/−^*^Δ*TA*^ also leads to mild oxidative stress triggered by DTT, but detailed mechanisms remain to be explored in future work.

## 5. Concluding Remarks

Hitherto, the overwhelming majority of redox researches have been focused principally on oxidative stress and antioxidant responses, owing to the relative rarity of workers on reductive stress and anti-reductant response. Herein, we found that DTT, as reductive stressor, stimulates distinct expression profiles of Nrf1 and Nrf2, along with their cognate target genes driven by antioxidant and/or electrophile response elements (AREs/EpREs). Their differential expression levels are also affected by the self-recovery (protective) ability of stimulated cells against such reductive drug cytotoxicity. By analyzing distinct expression levels of each gene in different genetic backgrounds of *WT*, *Nrf1α^−/−^*, *Nrf2^−/−^*^Δ*TA*^ and *caNrf2*^Δ*N*^ cell lines, we confirmed the distinct roles of Nrf1 and Nrf2 in mediating differential expression levels of both ARE-driven and UPR-target genes (Figure 6C). For example, XBP1 and Nrf1 are closely related, while HO-1, IRE1, CHOP and ATF4 genes are closely related to Nrf2. Although Nrf2 can serve as an upstream transcriptional regulator of Nrf1, it is inferred, according to the expression situation under this drug induction, that Nrf1 mainly regulates the expression of antioxidant and ER stress genes under normal state of cells, while Nrf2 mainly regulates the expression of intracellular genes under induced stress state. Overall, Nrf1 and Nrf2 are required for coordinated regulation of DTT-leading reductive stress response. However, DTT-stimulated expression of Nrf1-target proteasomal genes was still detected in *Nrf1α^−/−^* cells, but roughly unaffected by constitutive activation of Nrf2 (i.e., *caNrf2*^Δ*N*^) or its inactivation (i.e., *Nrf2^−/−^*^Δ*TA*^), demonstrating a requirement for such DTT-induced proteasomal genes regulated by an Nrf1/2-indepdent pathway. Lastly, the stability of Nrf1 and its transactivation activity are highlighted by the redox status of both the Cys342 and Cys640 residues located in its *trans*-acting glyco-domain and DNA-binding domain of this CNC-bZIP factor, respectively.

## Figures and Tables

**Table 1:** The primer pairs used for q-RT-PCR analysis.

Gene ID	Accession Number	Name	Forward Primers (5′-3′)	Reverse Primers (5′-3′)
60	NM_001101	*β*-actin	CATGTACGTTGCTATCCAGGC	CTCCTTAATGTCACGCACGAT
4779	NM_001330261	*Nrf1*	GAAGCCCACCAAGACCGAA	GCCTCTTCCTGTACACTGACC
4780	NM_001313903	*Nrf2*	ATATTCCCGGTCACATCGAGA	ATGTCCTGTTGCATACCGTCT
3162	NM_002133	*HO-1*	CAGAGCCTGGAAGACACCCTAA	AAACCACCCCAACCCTGCTAT
2729	NM_001197115	*GCLC*	TCAATGGGAAGGAAGGTGTGTT	TTGTAGTCAGGATGGTTTGCGA
2730	NM_001308253	*GCLM*	TGCTGTGTGATGCCACCAGA	CGCTTGAATGTCAGGAATGCTT
6888	NM_006755	*TALDO*	GGGCCGAGTATCCACAGAAG	GGCGAAGGAGAAGAGTAACG
1728	NM_001025434	*NQO1*	AAGAAGAAAGGATGGGAGGTGG	GAACAGACTCGGCAGGATACTG
2876	NM_001329503	*Gpx1*	CAGTCGGTGTATGCCTTCTCG	GAGGGACGCCACATTCTCG
2936	NM_001195102	*GSR*	CACGAGTGATCCCAAGCCC	CAATGTAACCTGCACCAACAATG
468	NM_182810	ATF4	CCCTTCACCTTCTTACAACCTC	TGCCCAGCTCTAAACTAAAGGA
22926	NM_007348	ATF6	CCCTCTCAGAAAACGAGCAAC	AGACAACTCTTCGCTTTGGAC
3309	NM_005347	BIP	AAAGCCACCAAGATGCTG	GCCTGCACTTCCATAGAGT
1649	NM_004083	*CHOP*	ACCAGCAGAGGTCACAAGCA	ATGACCACTCTGTTTCCGTT
2081	NM_001433	*IRE1*	AGTCTCTGCCCATCAACCTC	GCATTCCACCGGAGCTCTCG
9451	NM_001313915	*PERK*	TGCTTCTACAGCGTACCCAA	TCAATAAATCCGGCTCTCGT
7494	NM_001394000	*XBP1*	CACCCCTCCAGAACATCTCC	TGTCCAGAATGCCCAACAGG
5034	NM_000918	*P4HB*	ATCTTCATCGACAGCGACCACACCG	CGGTGTGGTCGCTGTCGATGAAGAT
5693	NM_001144932	*PSMB5*	ATCCGAGTCTCCAGTGACA	TCACCCCAAGAAACACAAGC
5694	NM_001270481	*PSMB6*	TCAAGAAGGAGGGCAGGTGT	GTAAAGTGGCAACGGCGAA
5695	NM_002799	*PSMB7*	CTGTGTCGGTGTATGCTCCA	TGCCAGTTTTCCGGACCTTT
4493	NM_175617	*MT1E*	ATGGACCCCAACTGCTCTTGCGCCA	ACAGCAGCTGCACTTCTCCGATG
4502	NM_005953	*MT2*	GTGGGCTGTGCCAAGTGT	CAAACGGTCACGGTCAGG

## Data Availability

All of the data is contained within the article and the supplementary materials.

## Supplementary Materials

The following supporting information can be downloaded at: https://www.mdpi.com/article/10.3390/antiox11081535/s1, Figure S1: An internal regulatory relationship between Nrf1 and Nrf2 in mediating reductive stress response to DTT.; Figure S2: Distinct time-dependent expression of Nrf1/2-mediated redox response genes to DTT in different cell lines; Figure S3: Distinct roles of Nrf1 and Nrf2 in the ER stress response induced by DTT; Figure S4: Nrf1 stability and trans-activity were determined by redox status of its Cys342 and Cys640; Figure S5: The overall regulation of differential expression genes in distinct cell lines after DTT treatment.
