# Supplementary material for: Distinct Roles of Nrf1 and Nrf2 in Monitoring the Reductive Stress Response to Dithiothreitol (DTT)
Source: Antioxidants (Basel). 2022 Aug 7;11(8):1535. doi: 10.3390/antiox11081535 (PMC9405177; doi:10.3390/antiox11081535)
Supplement: Supplementary file 1 [file antioxidants-11-01535-s001.zip › antioxidants-1825297-supplementary.pdf]

# Distinct roles of Nrf1 and Nrf2 in monitoring the reductive stress response to dithiothreitol (DTT)

Reziyamu Wufur[1,2], Zhuo Fan[1], Jianxin Yuan[1], Ze Zheng[1], Shaofan Hu[1,2], Guiyin Sun[2], and Yiguo Zhang[2,1]*
[1]The Laboratory of Cell Biochemistry and Topogenetic Regulation, College of Bioengineering and Faculty of Medical Sciences, Chongqing University, No. 174 Shazheng Street, Shapingba District, Chongqing 400044, China
[2]Chongqing University Jiangjin Hospital, School of Medicine, Chongqing University, No. 725 Jiangzhou Avenue, Dingshan Street, Jiangjin District, Chongqing 402260, China
*Correspondence to YZ: yiguozhang@cqu.edu.cn

**Supplemental materials:**

## Figure S1

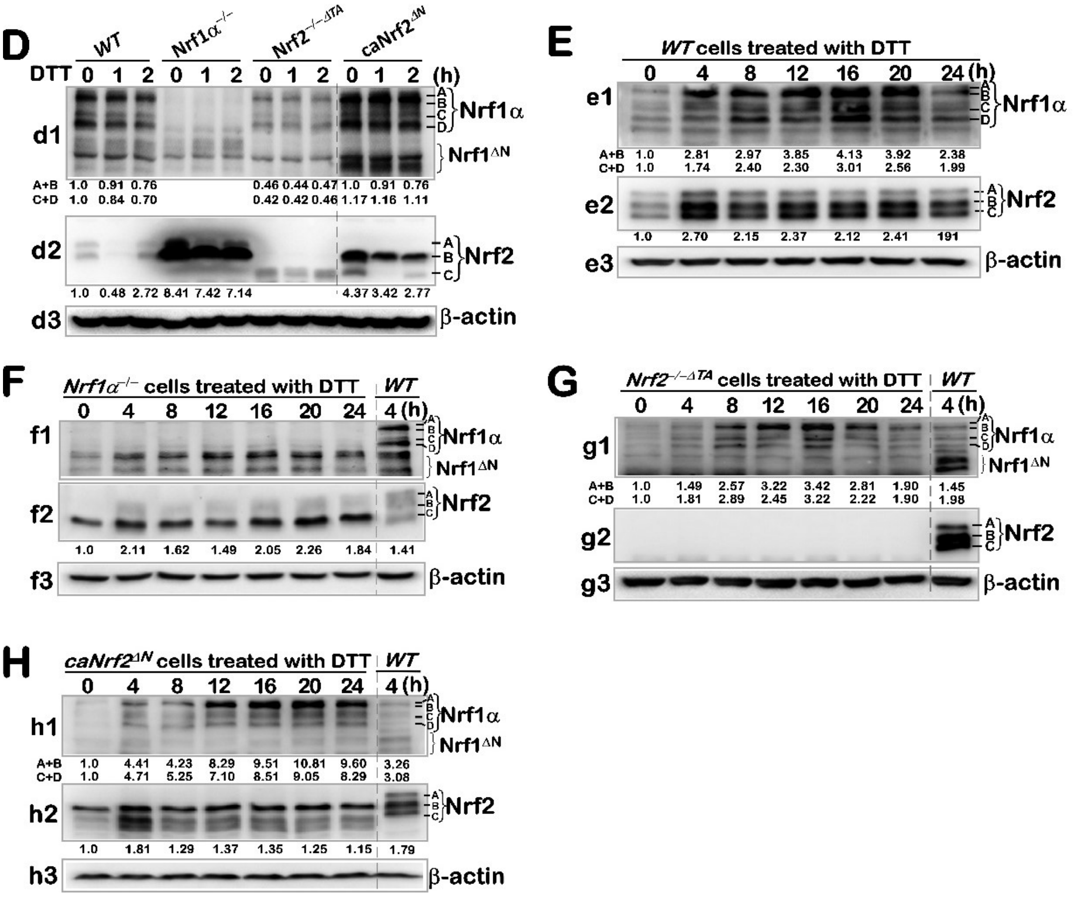

**Figure S1**. **An internal regulatory relationship between Nrf1 and Nrf2 in mediating reductive stress response to DTT.** The Nrf1/2 protein expression levels in four genotypic experimental cell lines (*WT, Nrf1α*$^{-/-}$, *Nrf2*$^{-/-\Delta TA}$ and *caNrf2*$^{\Delta N}$) intervention with 1 mM of DTT for short times (*i.e.* 0, 1, 2 h) **(D)** and long times (*i.e.* 0, 4, 8, 12, 16, 20, 24 h) **(E to H)**. All those protein-blotted bands were also qualified by Quantity One 4.5.2 software.

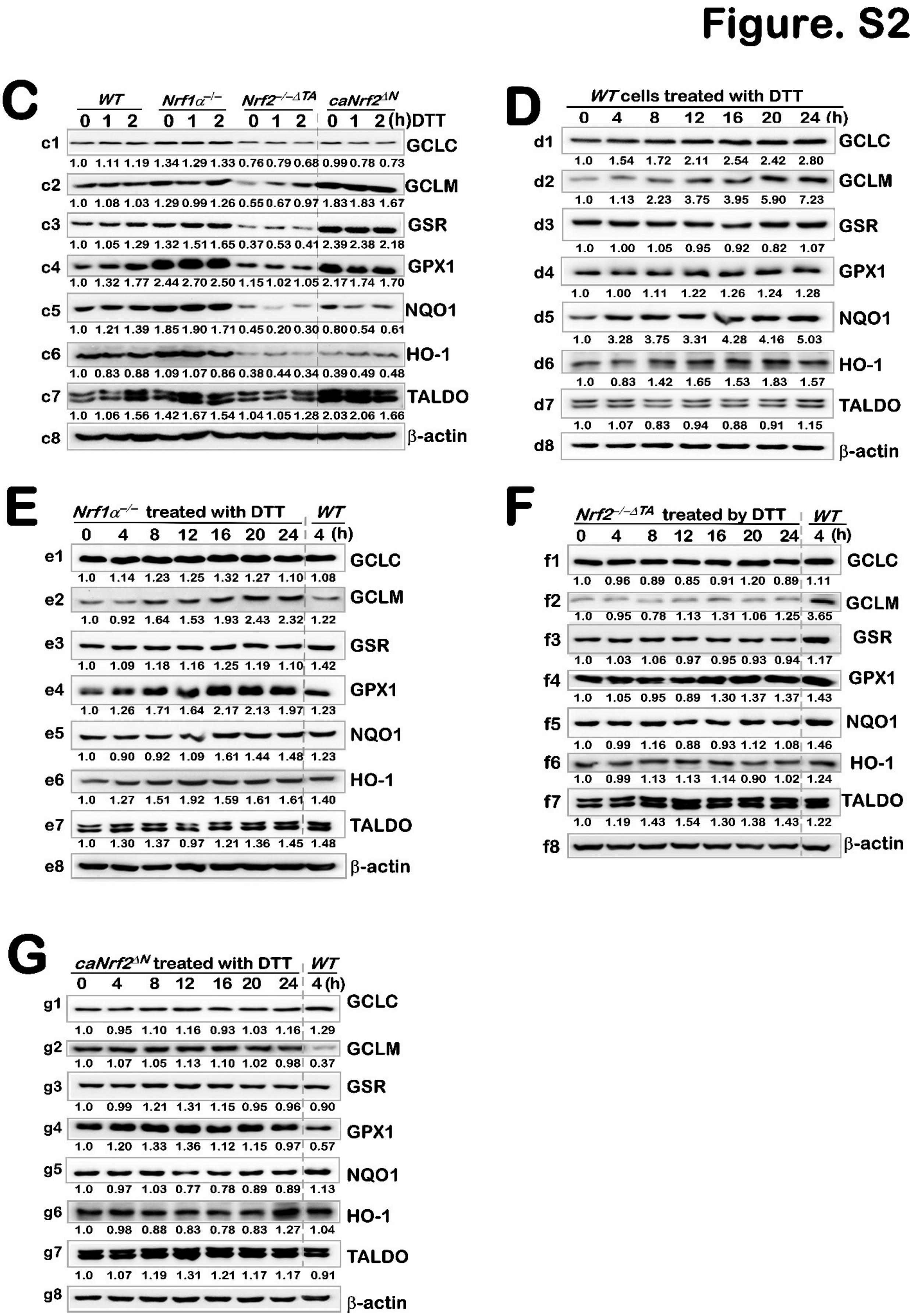


**Figure S2. Distinct time-dependent expression of Nrf1/2-mediated redox response genes to DTT in different cell**

**lines.** Different genotypic cell lines *WT, Nrf1α*$^{-/-}$, *Nrf2*$^{-/-\Delta TA}$ and *caNrf2*$^{\Delta N}$ were or were not treated with 1 mM DTT for short times (*i.e.* 0, 1, 2 h) **(C)** or long times (*i.e.* 0, 4, 8, 12, 16, 20, 24 h), before basal and DTT-inducible protein abundances of GCLC (*c1 to g1*), GCLM (*c2 to g2*), GSR (*c3, d3 to g3*), GPX1 (*c4 to g4*), NQO1 (*c5 to g5*), HO-1 (*c6 to g6*) and TALDO (*c7 to g7*) were determined by Western blotting with indicated antibodies, whilst *β*-actin served as a loading control. The intensity of those immunoblots was also quantified by the Quantity One 4.5.2 software.

# Figure S3

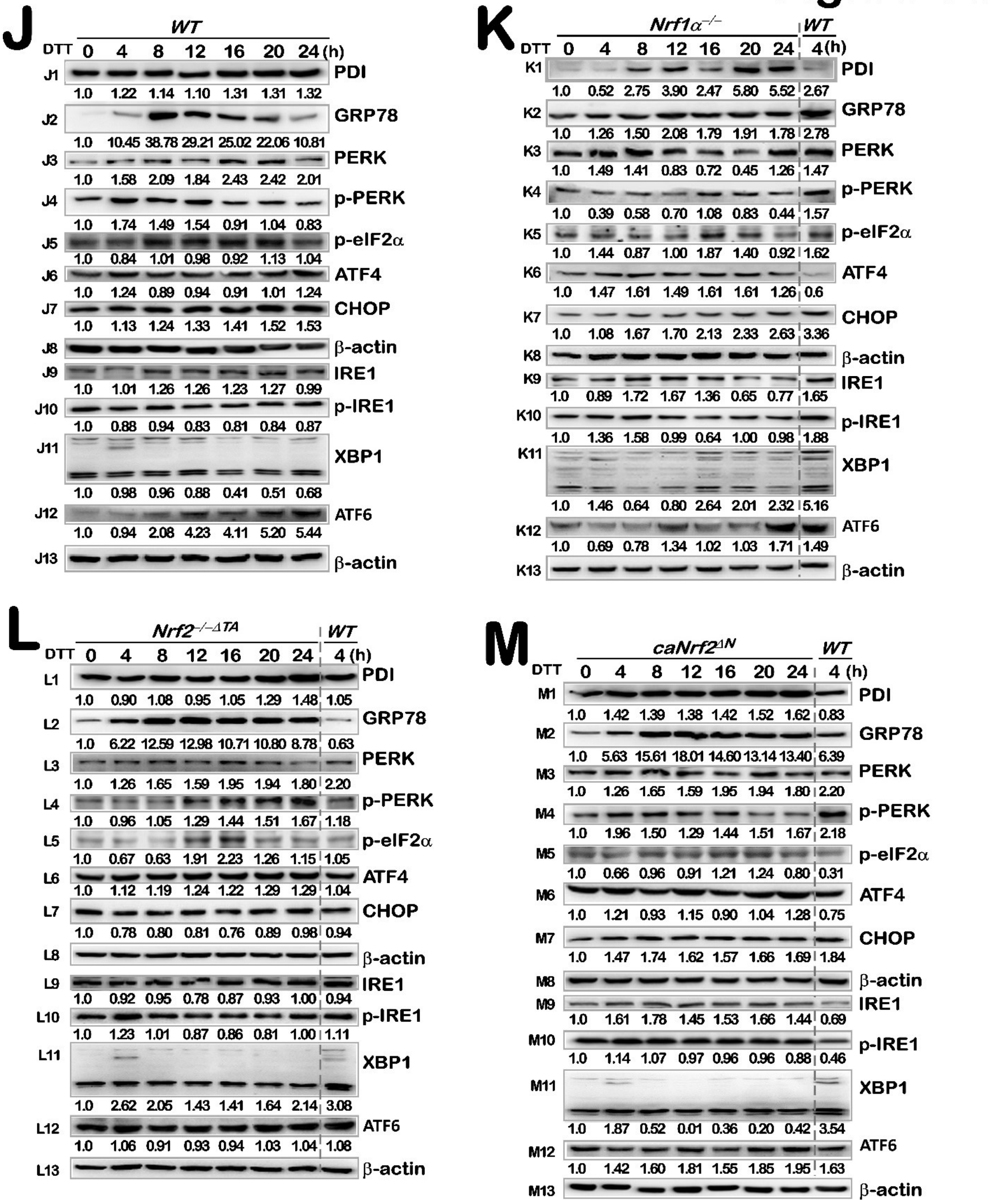


**Figure S3. Distinct roles of Nrf1 and Nrf2 in the ER stress response induced by DTT.** Four genotypic of *WT* **(J)**, *Nrf1α*$^{-/-}$ **(K)**, *Nrf2*$^{-/-\Delta TA}$ **(L)**, and *caNrf2*$^{\Delta N}$ **(M)** cell lines were or were not treated with 1mM DTT for distinct lengths of

time (*i.e.* 0, 4, 8, 12, 16, 20, 24 h), those inducible protein changes of PDI (*j1 to m1*), GRP78 (*j2 to m2*), PERK (*j3 to m3*), p-PERK (*j4 to m4*), p-eIF2$\alpha$ (*j5 tom5*), ATF4 (*j6 to m6*), CHOP (*j7 to m7*), IRE1 (*j9 to m9*), p-IRE1 (*j10 to m10*), XBP1 (*j11 to m11*)and ATF6 (*j12 to m12*) were determined by Western blotting with indicated antibodies, whilst $\beta$-actin served as a loading control. The intensity of those immunoblots, representing different protein expression levels, was also quantified by the Quantity One 4.5.2 software. The significant increases and significant decreases were represented with $\hat{}p < 0.05$ and $* p < 0.05$.

# Figure S4

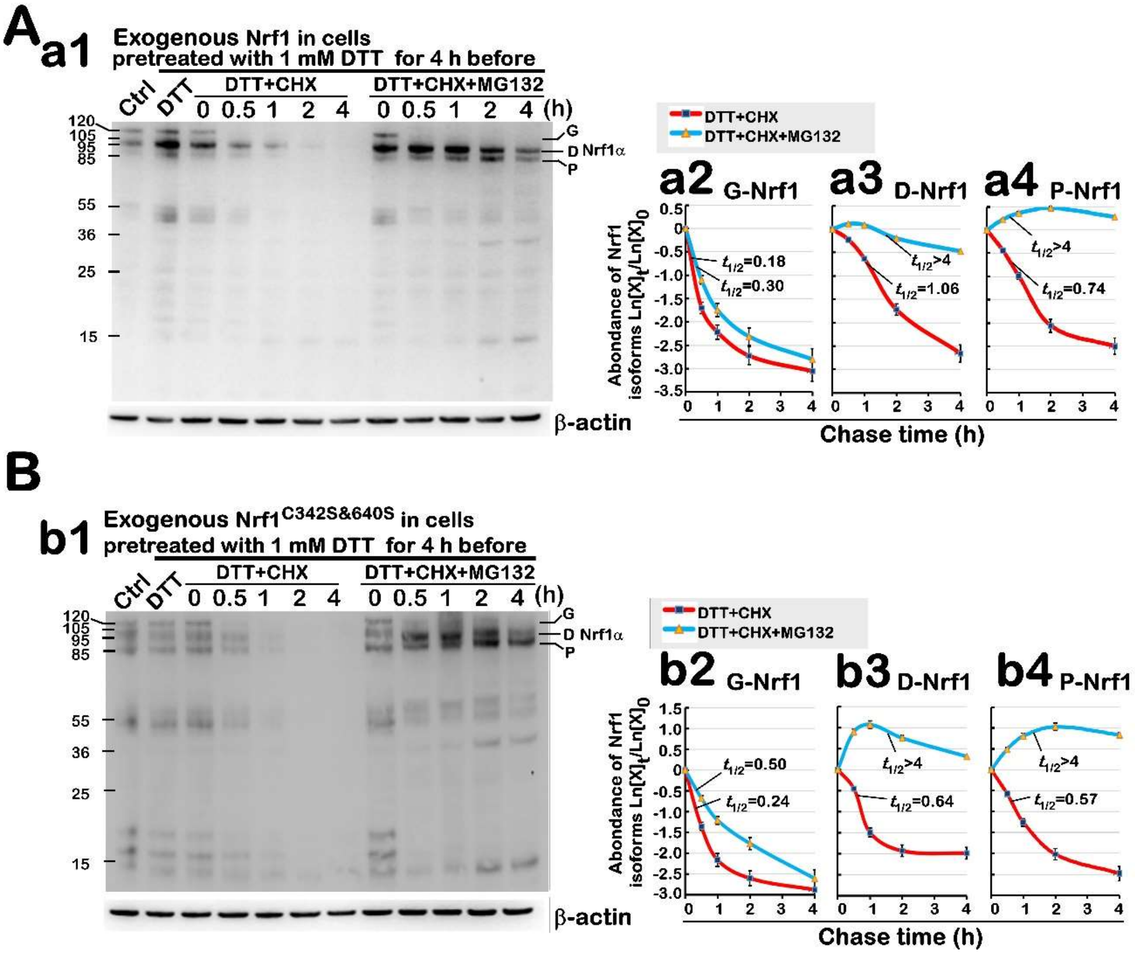


**Figure S4**. **Nrf1 stability and its trans-activity were determined by redox status of its Cys342 and Cys640.** **(A)** WT and **(B)** COS-1 cells were transfected with Nrf1$\alpha$ and its Nrf1$^{C342/640S}$ mutant plasmids as described in 'methods 2.6', pretreated with 1 mM DTT for 4 h, and then treated with CHX (50 μg/ml) and (or not) MG132 (10uM) for 0-4 h, respectively. And stability of exdogenous Nrf1$\alpha$ and its Nrf1$^{C342/640S}$ mutant proteins was showed by pulse-chase experiments (*a1*) (*b1*), and changes in its derived glycoprotein, deglycoprotein, processed protein abundances were represented by their respective turnover time-course curves (*a2, a3, a4*) (*b2, b3, b4*), respectively. The intensity of relevant immunoblots representing different protein expression levels was also quantified by the Quan-tity One 4.5.2 software. The resulting data were shown graphically, after being calculated by a formula of Ln ($[X]_t/[X]_0$), in which [X]t indicated a fold change (*mean* ± *SD*) in each of those examined protein expression levels at different times relative to corresponding controls measured at 0 h (*i.e.*, $[A]_0$), which were representative of at least three independent experiments.

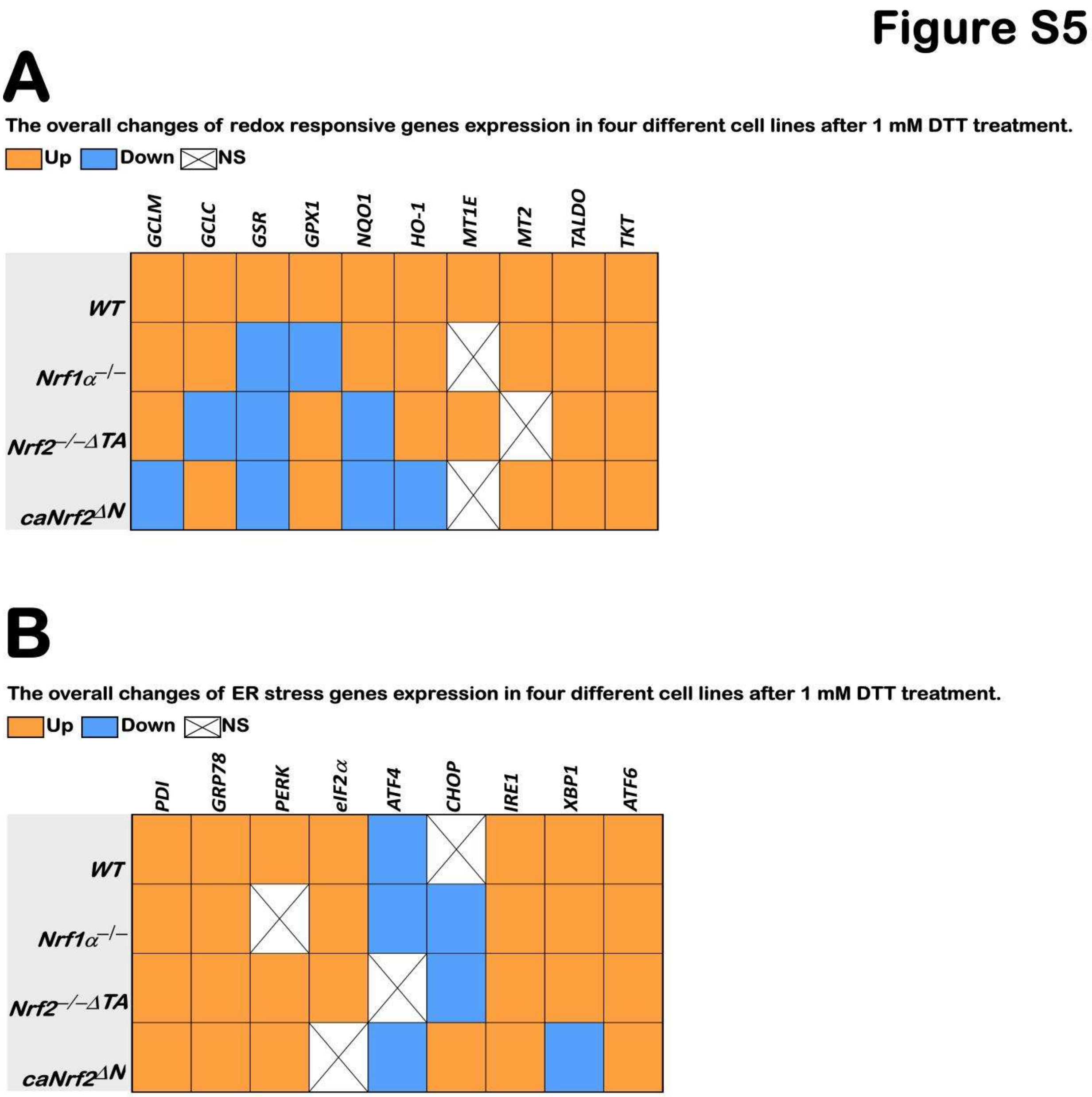


**Figure S5. The overall regulation of differential expression genes in distinct cell lines after DTT treatment. (A)** Up- and down- regulatory changes of examined gene expression related to redox in four genotypic cell lines after 1 mM DTT treatment. **(B)** Distinct regulatory changes in genes involved in the endoplasmic reticulum stress response upon treatment of four genotypic cell lines with 1 mM DTT treatment. The results were re-analyzed on the basis of the mRNA expression in **Figure 2** and **Figure 3**. First, sum up the expression amount of each gene in 4-24 h after DTT intervention and take the mean value, and then compare with $T_0$ among each cell line and plot at Graphpad prism 7.0. Among them, the orange box represents the genes with the overall significant increases, and the blue box represents the genes with the overall significant decreases after 1 mM DTT intervention, and those genes with no significant differences are the blank box. For the convenience of readers, this figure only shows the overall up- or down regulatory changes, but these differences between distinct cell lines or 1 mM DTT intervention time and target gene expression have also been in more detail, presented in **Figure 2 and 3**.
